# Chemical profiling and cytotoxic activity of the endophytic fungus *Epicoccum sorghinum* isolated from *Disynaphia filifolia*

**DOI:** 10.1007/s42770-026-01927-7

**Published:** 2026-04-29

**Authors:** Anderson Valdiney Gomes Ramos, Nathalia da Silva Malaco, Rodolfo Bento Balbinot, Drielli Rhiane Peres Colhado Areas, Francielli Alana Pereira Valeze, Camila Botin Francisco, Jesieli Beraldo Borrazzo, Andressa Domingos Polli, Julio Cesar Polonio, Celso Vataru Nakamura, João Alencar Pamphile, Ernani Abicht Basso, Maria Helena Sarragiotto, Debora Cristina Baldoqui

**Affiliations:** 1 Departamento de Química, Universidade Estadual de Maringá, Av. Colombo 5790, Maringá, Paraná 87020-900 Brazil; 2 Departamento de Biotecnologia, Genética e Biologia Celular, Universidade Estadual de Maringá, Av. Colombo 5790, Maringá, Paraná 87020-900 Brazil; 3 Programa de Pós-Graduação Em Ciências Biológicas, Universidade Estadual de Maringá, Av. Colombo 5790, Maringá , Paraná 87020-900 Brazil

**Keywords:** Asteraceae, Endophytic fungi, Cytotoxicity, Specialized metabolites, Tumor cells

## Abstract

Endophytic fungi are recognized as important sources of bioactive specialized metabolites. In this study, six compounds were isolated from cultures of endophytic fungus *Epicoccum sorghinum* (DF1) associated with *Disynaphia filifolia*, including a new aliphatic amide (**1**) identified as 2-hydroxy-*N*-(4’-oxohexan-2’-yl)-propanamide and other known compounds 2,3-dihydro-2-hydroxy-2,4-dimethyl-5-*trans*-propenylfuran-3-one (**2**), 2,3-dihydro-2-hydroxy-2,4-dimethyl-5-propylfuran-3-one (**3**), cyclo-*L*-Pro-*L*-Phe (**4**), cyclo-*L*-Pro-*L*-Tyr (**5**) and tetrahydroaltersolanol B (**6**). The structures were established by spectroscopic analysis and comparison with related compounds reported in the literature. The cytotoxic activities of the isolated compounds were evaluated against human prostate carcinoma (PC-3), cervical carcinoma (HeLa), and non-tumorigenic keratinocyte (HaCaT) cell lines. Among the tested compounds, cyclo-*L*-Pro-*L*-Phe showed the most pronounced cytotoxic effects against PC-3 and HeLa cells. The new amide derivative (**1**) exhibited weak activity toward PC-3 cells and no detectable activity against HeLa cells, while presenting low cytotoxicity toward HaCaT cells. The remaining compounds displayed weak to moderate cytotoxic activity, with PC-3 cells being the most sensitive overall. These findings contribute to the chemical characterization of metabolites produced by *E. sorghinum*, isolated for the first time from *D. filifolia*, and highlight its potential as a source of structurally diverse compounds with biological activity to be explored.

**Graphical Abstract:**

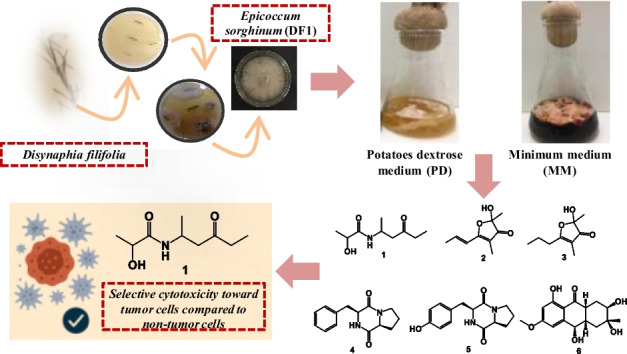

**Supplementary Information:**

The online version contains supplementary material available at 10.1007/s42770-026-01927-7.

## Introduction

Endophytic microorganisms inhabit the internal tissues of plants without causing disease and are increasingly recognized for their biotechnological and therapeutic potential [1, 2]. Among these, endophytic fungi are recognized as producers of structurally diverse specialized metabolites with a wide range of biological activities [3, 4]. Consequently, the isolation and characterization of metabolites from endophytic fungi represents a promising alternative source for the discovery of novel bioactive molecules of pharmaceutical interest [3, 5].

The genus *Epicoccum* (Didymellaceae) comprises filamentous fungi widely distributed in the environment, including soil, vegetation, and marine ecosystems. Species of this genus may occur as endophytes or plant pathogens and are frequently isolated from asymptomatic plant tissues [6–9]. Approximately 18 species of *Epicoccum* have been taxonomically recognized, with *Epicoccum nigrum* being the most extensively studied due to its ecological relevance and widespread occurrence [10].

Species of *Epicoccum* are known for producing a variety of bioactive specialized metabolites, including polyketides, diterpenes, and carotenoids, which display antimicrobial, antioxidant, antiviral and antiproliferative effects [8, 11, 12]. Additionally, non-toxigenic *Epicoccum* strains are promising sources of natural pigments for food industry applications, particularly carotenoids and polyketides like anthraquinones, naphthoquinones and azaphilones [13, 14].

*Epicoccum sorghinum*, formerly classified as *Phoma sorghina*, is widely recognized as one of the primary fungal species associated with sorghum grains [15]. Chemical studies involving endophytic strains of this species have reported the isolation of diphenyl esters [16], phenolic compounds [17], and bioactive exopolysaccharides (EPS) [18], indicating that its specialized metabolite profile is still underexplored.

Our research group has focused on the chemical investigation of endophytic fungi associated with Asteraceae species from the Campos Gerais region of Paraná, Brazil, an ecotone between the Atlantic Forest and Cerrado biomes [19]. In this context, *Epicoccum sorghinum* was isolated as an endophyte from *Disynaphia filifolia*, a native species whose aerial parts have previously been reported to contain tremetone as the major chemical constituent, along with other benzofuran derivatives, terpenes, phenols, and flavonoids [20].

Despite the recognized chemical diversity and biological relevance of endophytic fungi, microorganisms associated with *Disynaphia* species remain unexplored. Herein, we report the chemical characterization of metabolites produced by the endophytic fungus *Epicoccum sorghinum* DF1 isolated from *Disynaphia filifolia*, including the identification and structural elucidation of a previously undescribed aliphatic amide. In addition, the cytotoxic activities of isolated compounds were evaluated against two tumor cell lines and one non-tumoral cell line, contributing to the understanding of the chemical diversity and biological potential of endophytic fungi associated with this native plant species.

## Materials and methods

### Biological material

The endophyte DF1 was previously isolated from *Disynaphia filifolia* (Hassl.) R. M. King & H. Rob (Asteraceae) aerial parts and was retrieved from the Collection of Endophytic and Environmental Microorganisms at the Laboratory of Microbial Biotechnology (CMEA/LBIOMICUEM), Universidade Estadual de Maringá, Brazil. This endophyte was registered at the National System for the Management of Genetic Heritage and Associated Traditional Knowledge (SISGEN) under code AEE079A-17. *Disynaphia filifolia* is a native specimen, it was collected at Ponta Grossa city, Paraná State, Brazil (25° 05′ 16″ S, 50° 05′ 43″ W) in March 2016 and identified by Drª. Marta Regina Barrotto do Carmo. A voucher specimen was deposited at the herbarium at Universidade Estadual de Ponta Grossa (HUPG 22451). In addition, this plant was registered at SISGEN under code A6E6D08.

### DNA extraction, amplification and molecular identification

The identification of endophytic fungi was based on molecular and morphological analysis. The molecular identification was carried out using the internal transcript spacer regions (ITS), and partial sequence for beta-tubulin gene (TUB). The ITS region was amplified using primers ITS1 (5′-TCCGTAGGTGAACCTGCGG-3′) and ITS4 (5′-TCCTCCGCTTATTGATATGC-3′) [21]. For the TUB gene, primers T1 (5’-AACATGCGTGAGATTGTAAGT-3’) and Bt2b (5’-ACCCTCAGTGTAGTGACCCTTGGC-3’) were employed [22]. The amplification products were purified using a combination of two enzymes: Shrimp Alkaline Phosphatase (SAP) and Exonuclease I (EXO). The samples were sequenced by ACTGene Análises Moleculares LTDA (Ludwigbiotec). Fungal sequences were submitted to GenBank and compared with type sequences using BLAST. Multi-locus alignments were performed with MAFFT [23] in Geneious Prime v.2019.1.1. The best-fit evolutionary model was selected via MrModelTest v.2.3 [24]. Bayesian inference was conducted using MrBayes v.2.2.4 [25] (MCMC, 1 million generations; SD < 0.01), with posterior probabilities indicated at the nodes. The resulting phylogenetic tree was visualized and edited in FigTree v.1.4.2 [26].

The ITS and TUB DNA sequences was deposited in GenBank under accession numbers PX097382 and PX101957.

### Fermentation and extraction

Axenic maintenance of the endophytic DF1 strain was cultured and maintained using potato dextrose broth (PDB) (Acumedia®) and potato dextrose agar (PDA) (Acumedia®). The DF1 strain was subjected to two different cultivation conditions, using PDB and minimal medium (MM), at pH 6.8. The study hypothesis guiding this choice was that differences in nutrient composition, comparing a complex medium (PDB) with a chemically defined medium (MM), could modulate the expression of specialized metabolic pathways, potentially leading to qualitative variations in the metabolite profile.

The MM was prepared in the following concentrations: NaNO_3_ 60.00 g L^−1^, KH_2_PO_4_ 15.00 g L^−1^, KCl 5.00 g L^−1^, MgSO_4_·7H_2_O 5.00 g L^−1^, FeSO_4_·7H_2_O 0.01 g L^−1^, ZnSO_4_·7H_2_O 0.01 g L^−1^, CuSO_4_·7H_2_O 0.01 g L^−1^. At the time of use, the MM was diluted 1: 10 with distilled water and 10.00 g L^−1^ *D*-glucose was added [19].

Submerged cultivation of the endophytic was carried out to produce specialized metabolites. Three 10 mm diameter agar discs from the fungal strain grown on PDA were inoculated into 20 Erlenmeyer flasks (250 mL), each containing 100 mL of medium, totaling 2.0 L of culture volume. The pH of both media was adjusted to 6.8. Cultivation was carried out at 28 °C for 21 days under stationary conditions [27]. At the end of the incubation period, the mycelium was separated from the broth by vacuum filtration using a filter membrane, Büchner funnel and Kitasato. The resulting culture broth was extracted with ethyl acetate (3 × 150 mL). The organic phase was concentrated using a rotary evaporator (Tecnal TE-210) at 37 °C, yielding 80 mg and 55 mg of PDB and MM extracts, respectively. These values correspond to approximate yields of 40 mg L⁻^1^ for PDB and 27.5 mg L⁻^1^ for MM.

### General experimental procedures of metabolites isolation and identification

Chromatography separations were performed on silica gel (200–300 mesh; Qingdao Marine Chemical Plant Branch., China) or Sephadex LH-20 (100–200 mesh; Beijing Solarbio Technology Co., Ltd., China) chromatography columns (CC). Plates precoated with silica gel 60G or silica gel F254 (Rushan, Shandong Sun Desiccant Co., Ltd.) were used for thin layer chromatography (TLC). Visualization of the compounds on TLC was accomplished by UV irradiation at 254 and 366 nm and/or by spraying with H_2_SO_4_/anisaldehyde/acetic acid/methanol (5: 0.5: 10: 85 mL) solution followed by heating at 150 °C or Dragendorff’s solution. A Shimadzu LC-20AR HPLC was also used for analysis and isolation. For analysis, a Supelcosil LC-18 (25 cm × 4,6 mm, 5 µm). was used. The isolation was achieved on an Agilent semi-preparative Supelcosil LC-18 column (250 × 20 mm, 15 µm). UHPLC-ESIMS data were acquired on a Shimadzu Nexera X2 instrument system coupled with a Bruker IMPACT II mass spectrometer system equipped with an Electrospray ionization (ESI) source, in the positive and negative ion modes, quadrupole-time of flight (Q-Tof) analyzer, and multichannel plate detector. Optical rotations were acquired on a JASCO P-1010 polarimeter. NMR spectra were recorded on a Bruker Avance III HD spectrometer operating at 300 or 500 MHz (^1^H) and 75.5 or 125 MHz (^13^C) 75.5 MHz, using methanol-*d*_4_, DMSO-*d*_6_ and chloroform-*d* as solvents.

### Isolation and purification of compounds

The MM extract (55.0 mg) was fractionated using a silica gel column (flash chromatography), eluting with chloroform: methanol (100:0, 98:02, 95:05, 90:10, 85:15; 80:20; 30:70 and 50:50 v/v) gradient system to give eight fractions (DF1-MM-1 to DF1-MM-8). Subfraction DF1-MM-6 yielded compound **1** (4.3 mg). Subfraction DF1-MM-3 (7.0 mg) afforded the mixture of compounds **2** and **3** (7.0 mg).

PD extract (80.0 mg), which previously showed a TLC profile enriched in relatively polar compounds with strong retention on silica gel, was initially subjected to column chromatography in Sephadex LH-20 using methanol–water as the mobile phase in decreasing polarity gradient system (25:75, 50:50, 100:0), to give subfractions DF1-PD-1 to DF1-PD-12. Compound **2** (4.7 mg), previously isolated from the MM extract and identified based on its similar TLC profile, was reisolated from subfraction DF1-PD-2. The DF1-PD-3 subfraction was subjected to silica gel CC (flash chromatography) eluting with a gradient system hexane: ethyl acetate (50:50, 60:50, 0:100 v/v) to produce seven subfractions (DF1-PD-3–1 to DF1-PD-3–7). Subfractions DF1-PD-3–2 and DF1-PD-3–4 afforded the isolated compounds **4** (2.5 mg) and **5** (3.1 mg), respectively. The subfraction DF1-PD-5 (19.0 mg) was purified using semi-preparative reverse-phase HPLC equipped with a Shim-pack PREP-ODS C18 column (250 mm × 20 mm; 15 μm) using isocratic elution with MeOH:H_2_O (50:50 v/v) for 30 min, flow rate: 12 mL/min, to obtain compound **6** (2.4 mg, *t*_R_ = 11.2 min).

2-hydroxy-*N*-(4’-oxohexan-2’-yl)-propanamide (**1**): Yellow gum; [α]_D_^24^ + 45.5 (c 0.11, MeOH); ^1^H (300 MHz, CDCl_3_) and ^13^C (75.5 MHz, CDCl_3_) NMR data, see Table 1; HR-ESI–MS calculated for C_9_H_17_NO_3_ [M + H]^+^: 188.1281, found: 188.1275.

**Table 1:** ^1^H and ^13^C NMR data of compound **1**

Compound 1		
Position	**δ**_**H**_** (ppm, *J* = Hz)**^**a**^	**δ**_**C**_** (ppm)**^**a**^
1	1.40 (3H, d, 6.6)	21.4
2	4.17 (1H, q, 6.8)	68.3
3	-	173.7
1’	1.24 (3H, d, 6.73)	20.3
2’	4.32 (1H, m)	42.0
3’	2.65 (2H, dd, 5.2; 12.7)	47.4
4’	-	210.7
5’	2.45 (2H, m)	36.7
6’	1.04 (3H, t, 7.35)	7.6
NH	6.88 (1H, brs)	-

^a^(δ-ppm; 300 and 75.5 MHz; recorded in CDCl_3_)

### *In vitro *cytotoxic activity

The cytotoxic potential of compounds 1, 2, 4, 5, 6 and mixture of 2 + 3 were measured using the MTT colorimetric assay [28]. Cell viability was determined for the tumor cell lines HeLa (cervical adenocarcinoma, ATCC® CCL-2), PC-3 (prostate adenocarcinoma, ATCC® CRL-1435) and non-tumor HaCaT (immortalized human keratinocyte, CLS 300493). PC-3 cells were cultured in RPMI-1640 (pH 7.6), and HeLa and HaCaT cells were cultured in DMEM medium (pH 7.2). Additionally, the media were supplemented with *L*-glutamine and FBS at 10%, and incubated at 37 °C under a 5% CO_2_ atmosphere. The cells were plated at a density of 2.5 × 10^5^ cells mL^−1^ (equivalent to 2.5 × 10^4^ cells per well in 96-well plates, 100 µL/well). After 24 h, the cells were treated with increased concentrations (1.0 to 200 µg mL^−1^) of isolated compounds for 48 h. All stock solutions were prepared at 20 mg mL^−1^ in DMSO and diluted into culture medium to reach the working concentrations used in the assays. The final DMSO concentration in all experimental conditions did not exceed 1% (v/v). Doxorubicin was used as a reference drug treatment. After the treatment, media were carefully removed. Cells were washed twice with PBS (pH 7.2), subsequently 50 μL of MTT solution (2 mg mL^−1^ in PBS) was added to each well. The plate was incubated at 37 °C for 4 h. Then, the supernatant was removed, the formazan crystals were solubilized in DMSO and the absorbance reading was performed at 570 nm in a plate spectrophotometer (Power Wave XS, BioTek). Half-maximal inhibitory concentration (IC_50_) was determined as the concentration capable of reducing 50% of the optical density of the treated cells compared to the control, analysis performed by means of non-linear regression. The results were expressed as the mean ± standard deviation (SD) of at least three independent experiments.

### In-silico absorption, distribution, metabolism, excretion, and toxicity (ADMET) prediction

Due to its structural novelty, the pharmacokinetic properties of undescribed compound **1**, including absorption, distribution, metabolism, excretion and toxicity (ADMET), were predicted using the SWISSADME web server (http://www.swissadme.ch/) [29]. Key pharmaceutical parameters assessed included molecular weight, lipophilicity (Log P), solubility (Log S), blood–brain barrier (BBB) permeability, gastrointestinal (GI) absorption/oral bioavailability, and compliance with Lipinski’s Rule of Five (ROF). Additionally, CYP450 enzymes inhibition potential was evaluated through predictions generated by the SWISSADME web tool. The SMILES were used as an input.

### Statistical analysis

Statistical analyses were conducted using GraphPad Prism version 8.0.1 software (GraphPad, San Diego, CA, USA). Data normality was assessed using the Shapiro–Wilk test prior to statistical comparisons. As all datasets satisfied normal distribution criteria (*p* > 0.05), one-way analysis of variance (ANOVA) followed by Tukey's test was used to determine if there were statistically significant differences between treatments within each experiment. A 95% significance level (*p* < 0.05) was applied throughout the study.

## Results and discussion

### Identification of the fungal strain DF1

**Fig. 1:**
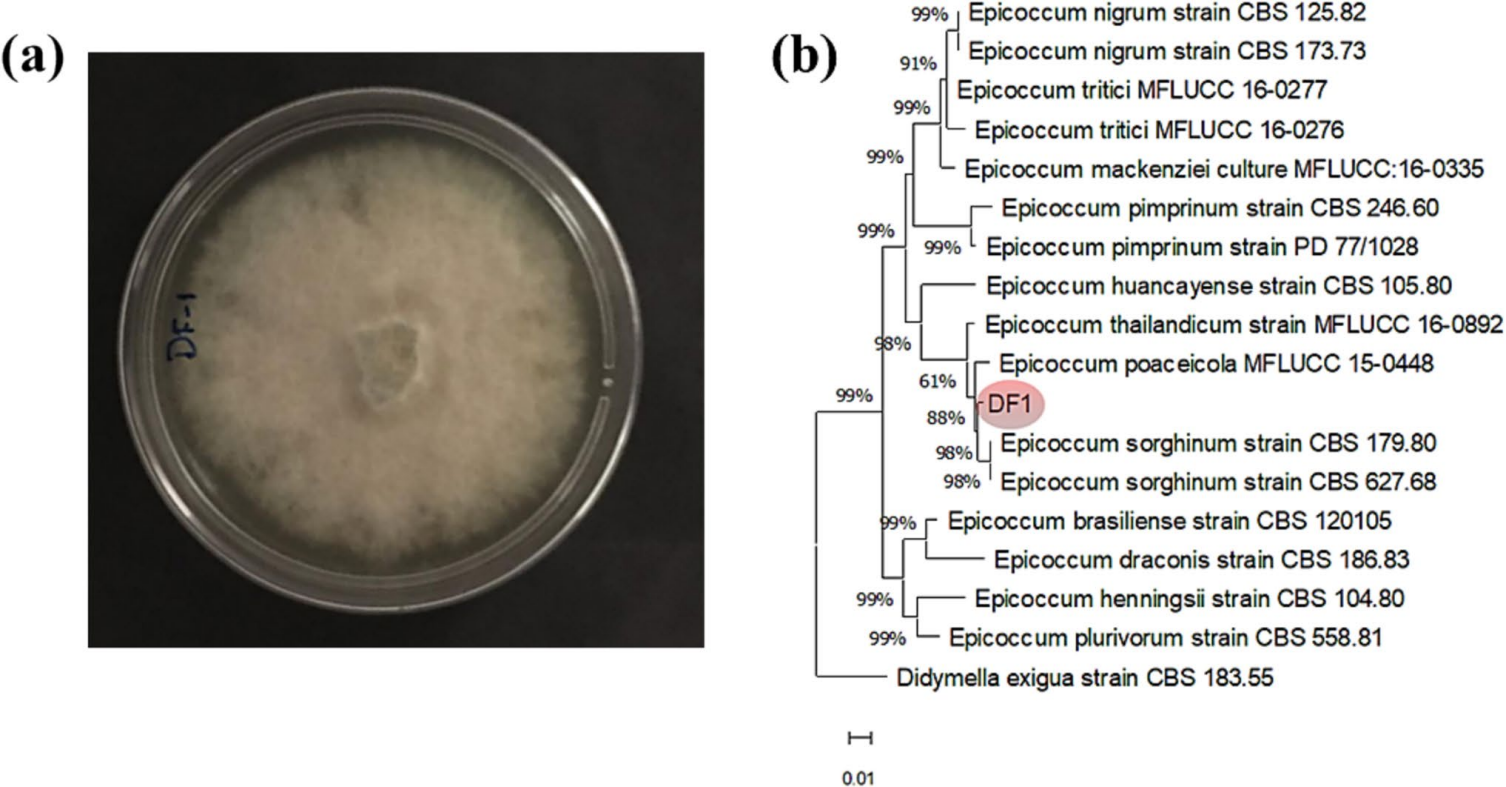
Identifying the species of strain DF1. (**a**): Conidiation morphology after 21 days of culture on a PDA plate at 28 °C (**b**): Phylogenetic consensus tree based on ITS1-5.8S-ITS2 gene sequences calculated by Bayesian inference. Bayesian probability was demonstrated at the nodes between each organism. The strain *Didymella exigua* CBS 183.55 was used as external group.

Morphological characteristics of strain DF1 on a PDA plate were observed after 21 days of growth at 28 °C and a phylogenetic tree was established based on ITS sequences (Fig. 1). When cultivated on PDA, it forms dense and fluffy mycelia with cottony and velvety textures, displaying a gray color and producing a reddish-brown pigment on the underside of the Petri dish. The ITS1-5.8 s-ITS2 gene of the endophytic fungus isolated from *D. filifolia* were sequenced and submitted to BLASTN in GenBank. The results indicate several species of *‘phoma-like’* belonging to the genus *Epicoccum* as the closest matches. Among the species analyzed, *Epicoccum sorghinum* strains, particularly CBS 627.68, exhibited 100% identity in the ITS region and 97% identity in the TUB gene. Phylogenetic analysis revealed that strain DF1 clusters closely with *E. sorghinum*, forming a well-supported clade with a bootstrap value of 98%.

### Purification and structure elucidation of compounds from Epicoccum sorghinum

**Fig. 2:**
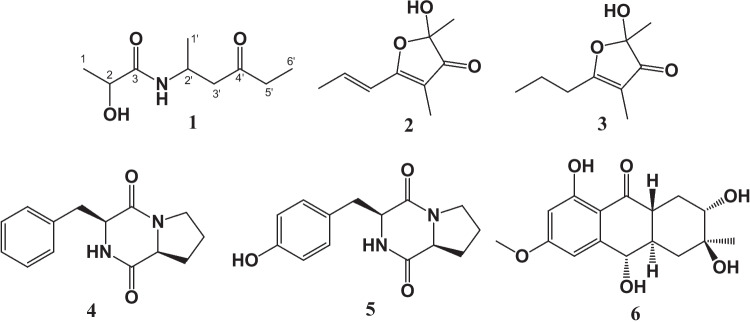
Chemical structures of compounds **1–6** isolated from *Epicoccum sorghinum*

From the ethyl acetate extract of a culture of the endophytic fungus *E. sorghinum* DF1, a undescribed compound denominated 2-hydroxy-*N*-(4’-oxohexan-2’-yl)-propanamide (**1**) together with five known compounds, including two furanones 2,3-dihydro-2-hydroxy-2,4-dimethyl-5-trans-propenylfuran-3-one (**2**) [30–32], 2,3-dihydro-2-hydroxy-2,4-dimethyl-5-propylfuran-3-one (**3**) [30], two diketopiperazines cyclo-*L*-Pro-*L*-Phe (**4**) [33, 34], cyclo-*L*-Pro-*L*-Tyr (**5**) [34], and a polyketide, tetrahydroaltersolanol B (**6**) [35, 36], were isolated (Fig. 2). The chemical structures were identified by NMR and ESI–MS/MS data and by comparison with the literature. Compound **1** was isolated exclusively from fermentation in minimal medium (MM). Compounds **4**, **5** and **6** were obtained only in potato dextrose medium (BD).

Compound **1** was obtained as an optically active yellow gum with a specific rotation of [α]_D_^24^ + 45.5 (c 0.11, MeOH), had a molecular formula C_9_H_17_NO_3_ as defined according to the HR-ESI–MS peak at *m/z* 188.1275 [M + H]^+^ (calcd. for C_9_H_17_NO_3_, 188.1281) (Fig. S12).

According to the characteristic signals of the ^1^H NMR spectrum (Table 1, Fig. S2-S3) revealed the presence of one oxymethynic proton δ_H_ 4.17 (1H, q, *J* = 6.8 Hz, H-2), one methynic δ_H_ 4.32 (1H, m, H-2’), two methylene [δ_H_ 2.65 (2H, dd, *J* = 5.2 Hz; 12.7 Hz, H-3’) and δ_H_ 2.45 (2H, m, H-5’)], and three methyl groups [δ_H_ 1.40 (3H, d, *J* = 6.6 Hz, H-1), δ_H_ 1.24 (3H, d, *J* = 6.7 Hz, H-1’), and δ_H_ 1.04 (3H, t, *J* = 7.4 Hz, H-6’)]. The ^13^C NMR data (Table 1; Fig. S4) and the HSQC spectrum (Fig. S8-S9) of **1** displayed 9 signals that could be assigned as two carbonyl carbons at δ_C_ 210.7 (C-4’) and 173.7 (C-3) characteristic of the ketone and amide groups, respectively. In addition to two methynic carbon at δ_C_ 42.0 (C-2’) and 68.3 (C-2), two methylene at δ_C_ 36.7 (C-5’) and 47.4 (C-3’), as well as three methyl carbon at δ_C_ 21.4 (C-1), 20.3 (C-1’) and 7.6 (C-6’). A broad singlet at δ_H_ 6.88 (NH), with no corresponding correlations in the HSQC spectrum (Fig. S8-S9), was attributed to hydrogen bonded to the nitrogen atom (NH) of the structure. This assignment was confirmed by the correlation observed in the COSY spectrum (Fig. S5-S7) between the signal at δ_H_ 4.32 (H-2’) and the signal at 6.88 (NH). Additionally, correlations were observed between the signals δ_H_ 4.17 (H-2) and δ_H_ 1.40 (H-1); δ_H_ 2.45 (H-5’) and δ_H_ 1.04 (H-6’); and the signal at δ_H_ 4.32 with the signals at δ_H_ 1.24 (H-1’) and δ_H_ 2.65 (H-3’).

**Fig. 3:**
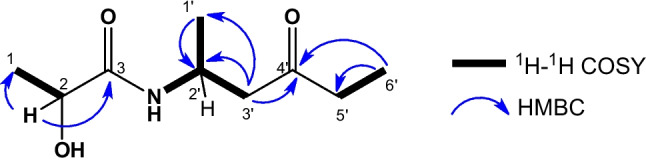
Key ^1^H–.^1^H COSY and HMBC correlations of compound **1**

The spin coupling system of H-2 (δ_H_ 4.17 1H, q, *J* = 6.8 Hz), along with the HMBC correlations (Fig. 3, Fig. S10-S11) of H-2 with C-1 (δ_C_ 21.4) and C-3 (δ_C_ 173.7), confirmed that the lactamide moiety was linked to the structure. In addition to the correlations between the signals at δ_H_ 2.65 (H-3’) with the carbons δ_C_ 42.0 (C-2’), 20.3 (C-1’) and 210.7 (C-4’), and the terminal methyl group at δ_H_ 1.04 (H-6’) with the carbons δ_C_ 36.7 (C-5’) and 210.7 (C-4’).

On this basis, compound **1** was identified as 2-hydroxy-*N*-(4′-oxohexan-2′-yl)-propanamide. Due to the limited amount of material, the absolute configuration could not be established. Although amide-containing metabolites have been reported from microbial sources [37–40], the compound **1** contains two stereogenic centers (C-2 and C-2′), and no closely related analogues with established absolute configurations were identified to support a reliable configurational assignment based on biosynthetic or spectroscopic comparison. In similar simple aliphatic amide systems, stereochemical outcomes may vary depending on the producing organism and biosynthetic pathway. If future studies enable the isolation of compound **1** in larger amounts, advanced optical stereochemical techniques could be employed to establish its absolute configuration. To the best of our knowledge, this study represents the first chemical characterization of this compound.

Compound **2** was previously isolated from the fungus *Stemphylium radicinum* [30], and has subsequently been identified in the endophytic fungus *Mollisia nigrescens*, associated with the leaves and stems of *Vaccinium angustifolium* [32]. It has also been reported in an endophytic *Penicillium* sp. isolated from *Taxus brevifolia* [31], and in the marine-derived fungus *Ascochyta salicorniae* from the green alga *Ulva* sp. [41]. The compound **3** was originally described by Grove (1971) [30] as the product of a chemical reduction of 2,3-dihydro-2-hydroxy-2,4-dimethyl-5-*trans*-propenylfuran-3-one (**2**). To the best of our knowledge, this is the first report of compound **3** being isolated as a natural product. Furthermore, this study provides the first report of the occurrence of these metabolites within the genus *Epicoccum*. Diketopiperazines **4** and **5** were previously isolated from *Epicoccum nigrum* strain M13 by Qader et al. (2021) [37]. However, this is the first report of these compounds in the species *E.* *sorghinum*. These cyclic dipeptides represent a prominent class of specialized metabolites that are widely produced by diverse fungal genera. Tetrahydroaltersolanol B (**6**) was previously isolated from the fungi *Alternaria solani* [35] and *Stemphylium botryosum* [42]. However, as far as we know, it is the first time to report of this compound within the genus *Epicoccum*.

### Cytotoxic activity

The compounds **1**, **2**, **4**, **5**, **6** and mixture of **2** + **3** were tested against tumoral cells of human prostate carcinoma (PC-3) and immortalized cervical carcinoma cells (HeLa). Cytotoxicity against a non-tumor immortalized human keratinocyte cells (HaCat) was also evaluated. Compound **3** was not isolated in pure form and, therefore, could not be subjected to cytotoxic evaluation due to insufficient available mass. The results obtained are presented in Table 2.

**Table 2:** Cytotoxicity of compounds isolated from *E. sorghinum* against different cell lines

Compounds	Cell lines/IC50 ± SD (µg/mL)			Selective index (SI)		
	HeLa*	PC-3*	HaCat**		HeLa	PC-3
**1**	> 200	110.58 ± 5.6^a^	135.49 ± 3.9^a^		ND	1.23
**2**	156.11 ± 19.5^a^	62.94 ± 1.4^b^	47.34 ± 5.7^b^		0.30	0.75
**2 + 3**	168.84 ± 15.2^a^	99.51 ± 8.2^ad^	70.48 ± 4.4^c^		0.42	0.71
**4**	24.65 ± 3.0^b^	37.91 ± 1.8^c^	39.44 ± 0.8^b^		1.60	1.04
**5**	167.12 ± 6.9^a^	105.31 ± 8.0^a^	46.37 ± 4.9^b^		0.28	0.44
**6**	> 200	91.24 ± 9.6^d^	67.67 ± 1.8^c^		ND	0.74
**Doxorubicin**	1.91 ± 0.17^c^	1.89 ± 0.11^e^	5.32 ± 0.58^d^		2.79	2.82

Data expressed as mean ± standard deviation of three independent experiments. One-way analysis of variance.

(ANOVA) tests were applied, with significant differences between means identified by Tukey’s post hoc test. In the same column, the values marked with the same lowercase letter are similar (*p* > 0.05), whereas the values with different lowercase letter are significantly different (*p* < 0.05). IC_50_: Half-maximal inhibitory concentration (50%); SD: Standard deviation (n = 3); *: tumor cell lines; **: non-tumor cell lines; HeLa: immortalized cervical carcinoma cells; PC-3: human prostate carcinoma; HaCat: immortalized human keratinocyte cells (non-tumor); (SI (Selective index) = IC₅₀ non-tumor cells/IC₅₀ tumor cells); ND: not determined.

Among the isolated compounds, cyclo-*L*-Pro-*L*-Phe (**4**) showed the highest cytotoxic activity, with IC_50_ values of 37.91 µg/mL against PC-3 and 24.65 µg/mL against HeLa cells. Although these values indicate measurable inhibitory effect, the activity may be considered moderate when compared with highly potent antitumor agents. Diketopiperazines (DKPs), a class of cyclic dipeptides, have been reported to exert antitumor effects by modulation of cell proliferation and apoptosis pathways [43–45]. Previous studies have reported that diketopiperazines isolated from the ethyl acetate extract of the cell-free filtrate of *Exiguobacterium acetylicum* exhibited anticancer potential against colorectal cancer HT-29 cells in vitro. Among them, cyclo-*L*-Pro-*L*-Phe demonstrated promising cytotoxic effects, supporting the potential biological relevance of proline-containing DKPs as antitumor agents [46].

The new compound 2-hydroxy-*N*-(4’-oxohexan-2’-yl)-propanamide (**1)** exhibited weak cytotoxicity against PC-3 cells (IC_50_ = 110.58 µg/mL) and no significant activity against HeLa cells at the highest concentration tested (200 µg/mL). These results indicate that compound **1** does not act as a potent cytotoxic agent under the experimental conditions employed. The furanone 2,3-dihydro-2-hydroxy-2,4-dimethyl-5-trans-propenylfuran-3-one (**2**) demonstrated moderate activity against the PC-3 (62.94 µg/mL) and weak activity against the HeLa cells (156.11 µg/mL). However, when compounds **2** and **3** were tested in mixture, a decrease in biological activity was observed, which was lower than the activity of compound **2** when tested individually. The remaining isolated compounds (**5** and **6**) exhibited weak to moderate cytotoxic effects. Tetrahydroaltersolanol B (**6**), previously isolated from *Stemphylium globuliferum*, has been reported as inactive against human chronic myeloid leukemia (K562) and human lung (A549) cancer cell lines when compared to altersolanol A. Structural modifications, including the reduction of carbonyl groups and the removal of hydroxyl substituents, appear to markedly reduce biological activity [47].

It is important to emphasize that the cytotoxic effects observed in this study were determined exclusively through in vitro viability assays, reflecting direct pharmacodynamic interactions of the compounds with tumour cells under controlled experimental conditions. No experiments were conducted to investigate specific molecular targets or signaling pathways. Moreover, pharmacokinetic parameters such as absorption, distribution, metabolism, and clearance were not experimentally evaluated, as these require in vivo models. The ADMET analysis performed for compound **1** represents a theoretical in silico prediction and should not be interpreted as experimentally validated pharmacokinetic data. Therefore, the present findings should be considered exploratory and restricted to cellular-level pharmacodynamic effects.

PC-3 cells were overall more sensitive to the tested compounds than HeLa cells. Although compound **1** showed limited inhibitory activity against tumor cells, it displayed low cytotoxicity toward non-tumor human keratinocyte cells (HaCaT), with an IC_50_ value of 135.49 µg/mL, suggesting a relatively low degree of general cytotoxicity. This profile may be advantageous for future investigations focusing on alternative biological targets or structural optimization. The other isolated compounds showed moderate cytotoxicity against HaCaT in the range of 39.4 µg/mL to 70.5 µg/mL.

The comparatively higher sensitivity observed in PC-3 cells may be partially attributed to intrinsic biological differences between tumor cell lines. PC-3 cells present distinct membrane lipid composition and metabolic characteristics, which can influence permeability and intracellular accumulation of moderately lipophilic compounds [48, 49]. In addition, differences in mitochondrial function and redox regulation reported in prostate cancer cells may affect susceptibility to bioactive metabolites [46]. These explanations remain speculative and warrant further investigation to clarify the basis of this differential sensitivity.

Physicochemical, pharmacokinetic and pharmacological parameters play a significant role in the discovery of novel drug candidates as many invented drugs fail in the development process. Therefore, an in silico ADMET evaluation of the undescribed compound **1** was performed using SwissADME [29] web tools (Table S1). Structurally, the molecule presented a number of hydrogen bond acceptors and hydrogen bond donors less than ten and five, respectively. In addition, the number of rotatable bonds in each molecule was less than ten, values that fall within the acceptable limits defined by Veber and Egan for compounds with good oral bioavailability and permeability (Topological Polar Surface Area, TPSA = 66.4 Å^2^. The molecule showed high gastrointestinal absorption, very good aqueous solubility, with estimated solubility up to 57.8 mg/mL (ESOL Log S = − 0.51), and moderate lipophilicity (consensus Log P = 0.48), indicating favorable oral bioavailability. This is consistent with the prediction of high gastrointestinal (GI) absorption, while the compound is not expected to cross the blood–brain barrier (BBB), which may be desirable depending on the therapeutic target. Although the compound presents favorable predicted pharmacokinetic properties, these characteristics do not necessarily correlate with in vitro cytotoxic potency. The relatively low lipophilicity and absence of structural motifs commonly associated with cytotoxic agents may partially explain its limited cytotoxic effects in vitro.

In terms of metabolism, compound **1** is predicted not to inhibit any of the major cytochrome P450 enzymes (CYP1A2, CYP2C19, CYP2C9, CYP2D6 and CYP3A4), minimizing the risk of metabolic interactions. No structural alerts (PAINS or Brenk) were identified, and the compound complies with Lipinski drug-likeness rule, suggesting a low risk of nonspecific toxicity, which is consistent with the reduced cytotoxicity, observed in HaCaT cells. Its synthetic accessibility score (2.22) indicates that the molecule is both synthetically tractable and suitable for medicinal chemistry optimization. Therefore, although compound **1** does not emerge as a strong cytotoxic lead, its ADMET profile supports its suitability as a chemically stable and biologically safe scaffold, potentially amenable to further structural modification or exploration of alternative biological activities.

## Conclusion

This study expands the chemical knowledge of the endophytic fungus *Epicoccum sorghinum* associated with *Disynaphia filifolia*, leading to the identification of six metabolites, including a previously undescribed aliphatic amide derivative, identified as 2-hydroxy-*N*-(4’-oxohexan-2’-yl)-propanamide. The biological evaluation demonstrated distinct cytotoxic profiles among the isolated compounds. The diketopiperazine cyclo-(*L*-Pro-*L*-Phe) exhibited the most pronounced cytotoxic effects, whereas the new amide showed low cytotoxicity toward non-tumor HaCaT cells and favorable in silico pharmacokinetic properties, suggesting its potential for further structural optimization or for the investigation of alternative biological activities. To the best of our knowledge, this is the first report describing an endophytic fungus isolated from a *Disynaphia* species. These findings expand the known chemical diversity of the genus *Epicoccum* and reinforce the potential of endophytic fungi associated with native plant species as a valuable source of structurally novel natural products.

## Supplementary Information

Below is the link to the electronic supplementary material.

Supplementary file1 (DOCX 935 KB)

## References

[1] White JF, Kingsley KL, Zhang Q, Verma R, Obi N, Dvinskikh S, Elmore MT, Verma SK, Gond SK, Kowalski KP (2019) Endophytic microbes and their potential applications in crop management. Pest Manag Sci 75:2558–2565. 10.1002/ps.5527 31228333 10.1002/ps.5527 PMC6771842

[2] Najjar AA (2025) Therapeutic potential of endophytic microbes: emphasizing both fungal and bacterial endophytes. Appl Microbiol 5:5. 10.3390/applmicrobiol5010005

[3] Enyi EO, Chigozie VU, Okezie UM, Udeagbala NT, Oko AO (2024) A review of the pharmaceutical applications of endophytic fungal secondary metabolites. Nat Prod Res 39(11):3295–3311. 10.1080/14786419.2024.2423036 39514834 10.1080/14786419.2024.2423036

[4] Gouda S, Das G, Sen SK, Shin H-S, Patra JK (2016) Endophytes: a treasure house of bioactive compounds of medicinal importance. Front Microbiol 7:1538. 10.3389/fmicb.2016.01538 27746767 10.3389/fmicb.2016.01538 PMC5041141

[5] Gupta A, Meshram V, Gupta M, Goyal S, Qureshi KA, Jaremko M, Shukla KK (2023) Fungal endophytes: microfactories of novel bioactive compounds with therapeutic interventions; a comprehensive review on the biotechnological developments in the field of fungal endophytic biology over the last decade. Biomolecules 13:1038. 10.3390/biom13071038 37509074 10.3390/biom13071038 PMC10377637

[6] Schulz B, Boyle C (2005) The endophytic continuum. Mycol Res 109:661–686. 10.1017/S095375620500273X 16080390 10.1017/s095375620500273x

[7] Lin ZY, Wei JJ, Zhang MQ, Xu SQ, Guo Q, Wang X, Wang JH, Chen BS, Que YX, Deng ZH (2015) Identification and characterization of a new fungal pathogen causing twisted leaf disease of sugarcane in China. Plant Dis 99:325–332. 10.1094/PDIS-06-14-0661-RE 30699701 10.1094/PDIS-06-14-0661-RE

[8] Braga RM, Padilla G, Araújo WL (2018) The biotechnological potential of *Epicoccum* spp.: diversity of secondary metabolites. Crit Rev Microbiol 44:759–778. 10.1080/1040841X.2018.1514364 30369284 10.1080/1040841X.2018.1514364

[9] Xing N, Luo Z, Cheng Y et al (2023) A new chlorogentisyl alcohol derivative from the marine-derived fungus *Epicoccum sorghinum*. Chem Nat Compd 59:666–669. 10.1007/s10600-023-04082-9

[10] Jayasiri SC, Hyde KD, Jones EBG, Jeewon R, Ariyawansa HA, Bhat JD, Camporesi E, Kang JC (2017) Taxonomy and multigene phylogenetic evaluation of novel species in *Boeremia* and *Epicoccum* with new records of *Ascochyta* and *Didymella* (Didymellaceae). Mycosphere 8(8):1080–1101. 10.5943/mycosphere/8/8/9

[11] Fatima N, Ismail T, Muhammad SA, Jadoon M, Ahmed S, Azhar S, Mumtaz A (2016) Epicoccum sp., an emerging source of unique bioactive metabolites. Acta Pol Pharm 73:13–21 27008796

[12] Abed RM (2021) Exploring fungal biodiversity of genus Epicoccum and their biotechnological potential. In: Abdel-Azeem AM, Yadav AN, Yadav N, Usmani Z (eds) Industrially Important Fungi for Sustainable Development. Fungal Biol. Springer, Cham, Switzerland. 10.1007/978-3-030-67561-5_7

[13] Mapari SAS, Thrane U, Meyer AS (2010) Fungal polyketide azaphilone pigments as future natural food colorants? Trends Biotechnol 28:300–307. 10.1016/j.tibtech.2010.03.004 20452692 10.1016/j.tibtech.2010.03.004

[14] Afroz Toma M, Rahman MH, Rahman MS et al (2023) Fungal pigments: carotenoids, riboflavin, and polyketides with diverse applications. J Fungi 9:454. 10.3390/jof9040454 10.3390/jof9040454 PMC10141606 37108908

[15] Oliveira RC, Goncalves SS, Silva CDC, Dilkin P, Madrid H, Correa B (2019) Polyphasic characterization of *Epicoccum sorghinum*: a tenuazonic acid producer isolated from sorghum grain. Int J Food Microbiol 292:1–7. 10.1016/j.ijfoodmicro.2018.12.004 30553177 10.1016/j.ijfoodmicro.2018.12.004

[16] Zhu J, Li Z, Lu H, Liu S, Ding W, Li J, Xiong Y, Li C (2021) New diphenyl ethers from a fungus *Epicoccum sorghinum* L28 and their antifungal activity against phytopathogens. Bioorg Chem 115:105232. 10.1016/j.bioorg.2021.105232 34371373 10.1016/j.bioorg.2021.105232

[17] Li CY, Chang CC, Tsai YH, El-Shazly M, Wu CC, Wang SW, Hwang TL, Wei CK, Hohmann J, Yang ZJ, Cheng YB, Wu YC, Chang FR (2020) Anti-inflammatory, antiplatelet aggregation, and antiangiogenesis polyketides from *Epicoccum sorghinum*: toward an understanding of its biological activities and potential applications. ACS Omega 5:11092–11099. 10.1021/acsomega.0c02489 32455230 10.1021/acsomega.0c01000 PMC7241018

[18] Balasubramaniam S, Sakthivel A, Ramesh K, Manisseeri C, Ganeshan S, Subramani M, Gnanajothi K (2024) Bioprospecting of exopolysaccharides from the endophytic fungi *Epicoccum sorghinum* AMFS4, for its structure, composition, bioactivities and application in seed priming. Nat Prod Res. 10.1080/14786419.2024.2380012 39049511 10.1080/14786419.2024.2380012

[19] Balbinot RB, de Oliveira JAM, Bernardi DI, Polli AD, Polonio JC, Cabral MRP, Zanqueta ÉB, Endo EH, Meneguello JE, Cardoso RF, Azevedo JL, Dias Filho BP, Nakamura TU, do Carmo MRB, Sarragiotto MH, Pamphile JA, Baldoqui DC (2021) *Chromolaena laevigata* (Asteraceae) as a source of endophytic non-aflatoxigenic *Aspergillus flavus*: chemical profile in different culture conditions and biological applications. Braz J Microbiol 52:1201–1214. 10.1007/s42770-021-00502-6 33929720 10.1007/s42770-021-00502-6 PMC8324641

[20] Chierici YR, Ramos AV, Areas DR, Battistella AC, Cottica SM, Tiuman TS, Mannochio-Russo H, Ruiz AL, Foglio MA, MR, Barrotto do Carmo, MH, Sarragiotto (2025) Phytochemical investigation and biological evaluation of *Disynaphia filifolia*: a novel source of tremetone in Asteraceae. Chem Biodivers 22(9):1–13. 10.1002/cbdv.202402696 10.1002/cbdv.202402696 PMC12435406 40222959

[21] White TJ, Bruns T, Lee S, Taylor J (1990) Amplification and direct sequencing of fungal ribosomal RNA genes for phylogenetics. In: Innis MA, Gelfand DH (eds) PCR protocols: a guide to methods and applications, pp 315–322.

[22] Carbone I, Kohn LA (1999) A method for designing primer sets for speciation studies in filamentous ascomycetes. Mycologia 91:553–556. 10.1080/00275514.1999.12061051

[23] Katoh K, Toh H (2008) Recent developments in the MAFFT multiple sequence alignment program. Brief Bioinform 9:286–298. 10.1093/bib/bbn013 18372315 10.1093/bib/bbn013

[24] Nylander JAA (2004) MrModeltest Version 2. Evolutionary Biology Centre, Uppsala University, Uppsala

[25] Ronquist F, Teslenko M, Mark PVD, Ayres DL, Darling A, Höhna S, Larget B, Liu L, Suchard MA, Huelsenbeck JP (2012) Mrbayes 3.2: efficient Bayesian phylogenetic inference and model choice across a large model space. Syst Biol 61:539–542. 10.1093/sysbio/sys029 22357727 10.1093/sysbio/sys029 PMC3329765

[26] Rambaut A (2009) FigTree version 1.3.1. Computer program and documentation distributed by the author. http://tree.bio.ed.ac.uk/software/. Accessed 12 April 2025.

[27] Polli AD, Ribeiro MAS, Garcia A, Polonio JC, Santos CM, Silva AA, Orlandelli RC, Castro JC, Abreu-Filho BA, Cabral MRP, Sarragiotto MH, Pamphile JA, Azevedo JL (2020) Secondary metabolites of *Curvularia* sp. G6-32, an endophyte of *Sapindus saponaria*, with antioxidant and anticholinesterasic properties. Nat Prod Res 35(21):4148–4153. 10.1080/14786419.2020.1739681 32174195 10.1080/14786419.2020.1739681

[28] Mossmann T (1983) Rapid colorimetric assay for cellular growth and survival: application to proliferation and cytotoxicity assays. J Immunol Methods 65:55–63. 10.1016/0022-1759(83)90303-4 6606682 10.1016/0022-1759(83)90303-4

[29] Daina A, Michielin O, Zoete V (2017) Swissadme: a free web tool to evaluate pharmacokinetics, drug-likeness and medicinal chemistry friendliness of small molecules. Sci Rep 7:42717. 10.1038/srep42717 28256516 10.1038/srep42717 PMC5335600

[30] Grove JF (1971) Metabolic products of *Stemphylium radicinum*. IV. Minor products. Journal of the Chemical Society C: Organic. 10.1039/J39710002261 10.1039/j39710002261 5170055

[31] Stierle DB, Stierle AA, Ganser B (1997) New phomopsolides from a *Penicillium* sp. J Nat Prod 60:1207–1209. 10.1021/np970338f 9392888 10.1021/np970338f

[32] Ibrahim A, Sorensen D, Jenkins HA, Ejim L, Capretta A, Sumarah MW (2017) Epoxynemanione A, nemanifuranones A-F, and nemanilactones A–C, from *Nemania serpens*, an endophytic fungus isolated from Riesling grapevines. Phytochemistry 140:16–26. 10.1016/j.phytochem.2017.04.009 28441516 10.1016/j.phytochem.2017.04.009

[33] Furtado NAJC, Pupo MT, Carvalho I, Campo VL, Duarte MCT, Bastos JK (2005) Diketopiperazines produced by an *Aspergillus fumigatus* Brazilian strain. J Braz Chem Soc 16:1448–1453. 10.1590/S0103-50532005000800026

[34] Jinendiran S, Teng W, Dahms HU, Liu W, Ponnusamy VK, Chiu CC, Kumar BSD, Sivakumar N (2020) Induction of mitochondria-mediated apoptosis and suppression of tumor growth in zebrafish xenograft model by cyclic dipeptides identified from *Exiguobacterium acetylicum*. Sci Rep 10:13721. 10.1038/s41598-020-70516-x 32792514 10.1038/s41598-020-70516-x PMC7426938

[35] Stoessl A, Stothers JB (1983) Tetrahydroaltersolanol B, a hexahydroanthronol from *Alternaria solani*. Can J Chem 61:378–382. 10.1139/v83-068

[36] Zheng CJ, Shao CL, Guo ZY, Chen JF, Deng DS, Yang KL, Chen YY, Fu XM, She ZG, Lin YC, Wang CY (2012) Bioactive hydroanthraquinones and anthraquinone dimers from a soft coral-derived *Alternaria* sp. fungus. J Nat Prod 75:189–197. 10.1021/np200766d 22276679 10.1021/np200766d

[37] Qader MM, Hamed AA, Soldatou S, Abdelraof M, Elawady ME, Hassane ASI, Belbahri L, Ebel R, Rateb ME (2021) Antimicrobial and antibiofilm activities of the fungal metabolites isolated from the marine endophytes *Epicoccum nigrum* M13 and *Alternaria alternata* 13A. Mar Drugs 19:232 33924262 10.3390/md19040232 PMC8074750

[38] Chen Y, Wang H, Sang Z, Qiu K, Wei S, Duan F, Zou Z, Tan H (2023) Two new secondary metabolites isolated from the fungus *Penicillium virgatum* T49-A. Fitoterapia 168:105513. 10.1016/j.fitote.2023.105513 37084850 10.1016/j.fitote.2023.105513

[39] Chen J, Xu AR, Shi BB, Zhang XF, Liu JK, Feng T (2025) Trichothecroamides A–F, antifungal amides from potato-associated fungus *Trichothecium crotocinigenum*. Phytochemistry 234:114438. 10.1016/j.phytochem.2025.114438 39955043 10.1016/j.phytochem.2025.114438

[40] Li F, Gu S, Zhang S, Mo S, Guo J, Hu Z, Zhang Y (2023) Three new amide derivatives from the fungus *Alternaria brassicicola*. Nat Prod Bioprospect 13:28. 10.1007/s13659-023-00391-2 37695377 10.1007/s13659-023-00391-2 PMC10495297

[41] Osterhage C, Kaminsky R, König GM, Wright AD (2000) Ascosalipyrrolidinone A, an antimicrobial alkaloid, from the obligate marine fungus *Ascochyta salicorniae*. J Org Chem 65:6412–6417. 10.1021/jo000307g 11052082 10.1021/jo000307g

[42] Aly AH, Debbab A, Edrada-Ebel RA, Müller WEG, Kubbutat MHG, Wray V, Ebel R, Proksch P (2010) Protein kinase inhibitors and other cytotoxic metabolites from the fungal endophyte *Stemphylium botryosum* isolated from *Chenopodium album*. Mycosphere 1:153–162

[43] Bojarska J, Breza M, Remko M, Czyz M, Gajos-Michniewicz A, Zimecki M, Kaczmarek K, Madura ID, Wojciechowski JM, Wolf WM (2022) Structural and biofunctional insights into the cyclo(Pro-Pro-Phe-Phe-) scaffold from experimental and in silico studies: melanoma and beyond. Int J Mol Sci 23:7173. 10.3390/ijms23137173 35806175 10.3390/ijms23137173 PMC9266943

[44] Hernández-Padilla L, Vázquez-Rivera D, Sánchez-Briones L et al (2017) The antiproliferative effect of cyclodipeptides from *Pseudomonas aeruginosa* PAO1 on HeLa cells involves inhibition of phosphorylation of Akt and S6k kinases. Molecules 22:1024. 10.3390/molecules22061024 28632179 10.3390/molecules22061024 PMC6152764

[45] Vázquez-Rivera D, González O, Guzmán-Rodríguez J et al (2015) Cytotoxicity of cyclodipeptides from *Pseudomonas aeruginosa* PAO1 leads to apoptosis in human cancer cell lines. Biomed Res Int 2015:197608. 10.1155/2015/197608 25821788 10.1155/2015/197608 PMC4363556

[46] Sharma LK, Fang H, Liu J, Vartak R, Deng J, Bai Y (2011) Mitochondrial respiratory complex I dysfunction promotes tumorigenesis through ROS alteration and AKT activation. Hum Mol Genet 20:4605–4616. 10.1093/hmg/ddr395 21890492 10.1093/hmg/ddr395 PMC3209831

[47] Jinendiran S, Teng W, Dahms HU et al (2020) Induction of mitochondria-mediated apoptosis and suppression of tumor growth in zebrafish xenograft model by cyclic dipeptides identified from *Exiguobacterium acetylicum*. Sci Rep 10:13721. 10.1038/s41598-020-70516-x 32792514 10.1038/s41598-020-70516-x PMC7426938

[48] van Meer G, Voelker DR, Feigenson GW (2008) Membrane lipids: where they are and how they behave. Nat Rev Mol Cell Biol 9:112–124. 10.1038/nrm2330 18216768 10.1038/nrm2330 PMC2642958

[49] Nicolson GL (2014) The fluid-mosaic model of membrane structure: still relevant after more than 40 years. Biochim Biophys Acta 1838:1451–1466. 10.1016/j.bbamem.2013.10.019 24189436 10.1016/j.bbamem.2013.10.019

