# Supplementary material for: Chemical profiling and cytotoxic activity of the endophytic fungus Epicoccum sorghinum isolated from Disynaphia filifolia
Source: Braz J Microbiol. 2026 Apr 29;57(1):129. doi: 10.1007/s42770-026-01927-7 (PMC13129151; doi:10.1007/s42770-026-01927-7)
Supplement: Supplementary file 1 — Supplementary file1 (DOCX 935 KB) [file 42770_2026_1927_MOESM1_ESM.docx]

**Chemical profiling and cytotoxic activity of the endophytic fungus *Epicoccum sorghinum* isolated from *Disynaphia filifolia***

Anderson Valdiney Gomes Ramos^1^, Nathalia da Silva Malaco^1^, Rodolfo Bento Balbinot^3^, Drielli Rhiane Peres Colhado Areas^1^, Francielli Alana Pereira Valeze^1^, Camila Botin Francisco^1^, Jesieli Beraldo Borrazzo^2^, Andressa Domingos Polli^2^, Julio Cesar Polonio^2^, Celso Vataru Nakamura^3^, João Alencar Pamphile^2^, Ernani Abicht Basso^1^, Maria Helena Sarragiotto^1^, Debora Cristina Baldoqui^1^*

*^1^Departamento de Química, Universidade Estadual de Maringá, Av. Colombo 5790, 87020-900, Maringá, Paraná, Brazil.*

*^2^Departamento de Biotecnologia, Genética e Biologia Celular, Universidade Estadual de Maringá, Av. Colombo 5790, 87020-900, Maringá, Paraná, Brazil.*

*^3^Programa de Pós-Graduação em Ciências Biológicas, Universidade Estadual de Maringá, Av. Colombo 5790, 87020-900, Maringá, Paraná, Brazil.*

**

**Figure S1.** Chemical structures of compounds **1–6** isolated from *Epicoccum sorghinum*

**2-hydroxy-*N*-(4’-oxohexan-2’-yl)-propanamide** **(1):** ^1^H NMR (500 MHz, CDCl_3_): δ 6.88 (1H, brs, NH), 4.32 (1H, m, H-2’), 4.17 (1H, q, *J =* 6.8 Hz, H-2), 2.65 (2H, dd, *J =* 5.2 and 12.7 Hz, H-3’), 2.45 (2H, m, H-5’), 1.40 (3H, d, *J =* 6.6 Hz, H-1), 1.24 (3H, d, *J =* 6.73 Hz, H-1’), 1.04 (3H, t, *J =* 7.35 Hz, H-6’). ^13^C NMR (125 MHz, CDCl_3_): δ 210.7 (C-4’), 173.7 (C-3), 68.3 (C-2), 47.4 (C-3’), 42.0 (C-2’), 36.7 (C-5’), 21.4 (C-1), 20.3 (C-1’), 7.6 (C-6’) (**Table 1**).

**2,3-dihydro-2-hydroxy-2,4-dimethyl-5-*trans*-propenylfuran-3-one (2):** ^1^H NMR (500 MHz, CDCl_3_): δ 6.86 (1H, dq, *J =* 15.6 and 6.8 Hz, H-7), 6.36 (1H, dd, *J =* 15.6 and 1.5 Hz, H-6), 1.98 (3H, dd, *J =* 6.8 and 1.5 Hz, H-8), 1.73 (3H, s, H-9), 1.55 (3H, s, H-1). ¹³C NMR (125 MHz, CDCl₃): δ 202.3 (C-3), 176.7 (C-5), 139.6 (C-6), 119.0 (C-7), 107.0 (C-4), 102.0 (C-2), 22.3 (C-1), 19.1 (C-8), 5.5 (C-9).

**2,3-dihydro-2-hydroxy-2,4-dimethyl-5-propylfuran-3-one (3):** ^1^H NMR (500 MHz, CDCl_3_): δ 2.49 (2H, t, *J =* 7.5 Hz, H-6), 1.70 (2H, m, H-7), 1.67 (3H, s, H-9), 1.52 (1H, s, H-1), 0.98 (3H, t, *J =* 7.5 Hz, H-7). ¹³C NMR (125 MHz, CDCl₃): δ 202.8 (C-3), 187.5 (C-5), 107.8 (C-4), 101.8 (C-2), 30.8 (C-6), 22.2 (C-1), 19.1 (C-7), 13.8 (C-8), 5.6 (C-9).

**Cyclo-*L*-Pro-*L*-Phen (4):** ^1^H NMR (500 MHz, CDCl_3_): δ 7.22–7.29 (5H, m, H-2’, H-3’, H-4’, H-5’ e H-6’), 4.44 (1H, t, *J =* 3.7 Hz, H-9), 4.06 (1H, ddd, *J =* 10.2, 6.6 and 1.5 Hz, H-6), 3.54 (1H, m, H-3), 3.36 (1H, m, H-3), 3.15 (2H, m, H-10), 2.09 (1H, m, H-5), 1.80 (2H, m, H-4), 1.25 (1H, m, H-5). ¹³C NMR (125 MHz, CDCl₃): δ 170.9 (C-7), 166.9 (C-1), 137.3 (C-1'), 131.0 (C-3', C-5’), 129.4 (C-2', C-6'), 128.0 (C-4'), 60.0 (C-6), 57.6 (C-9), 45.9 (C-3), 38.2 (C-10), 29.4 (C-5), 22.7 (C-4).

**Cyclo-*L*-Pro-*L*-Tyr (5):** ^1^H NMR (500 MHz, CDCl_3_): δ 7.04 (2H, d, *J =* 8.4 Hz, H-6' and H-2’), 6.70 (2H, d, *J =* 8.4 Hz, H-5' and H-3’), 4.36 (1H, t, *J =* 4.6 Hz, H-9), 4.06 (1H, m, H-6), 3.35–3.54 (2H, m, H-3), 3.05 (2H, dq, *J =* 4.6 and 13.8 Hz, H-10), 2.09 (1H, m, H-5) and 1.23 (1H, m, H-5), 1.79 (2H, m, H-4). ¹³C NMR (125 MHz, CDCl₃): δ 170.7 (C-7), 166.9 (C-1), 157.6 (C-4'), 132.1 (C-2'), 129.4 (C-6'), 127.6 (C-1'), 116.2 (C-5', C-3’), 45.9 (C-3), 29.3 (C-5), 22.6 (C-4).

**Tetrahydroaltersolanol B (6)** ¹H NMR (500 MHz, CD₃OD): δ 6.77 (1H, dd, *J =* 2.3 and 1.2 Hz, H-8), 6.32 (1H, d, *J =* 2.3 Hz, H-6), 4.37 (1H, d, *J =* 11.2 Hz, H-9), 3.84 (3H, s, H-12), 3.46 (1H, dd, *J =* 4.6 and 11.7 Hz, H-3), 2.41 (1H, ddd, *J =* 3.8, 12.0 and 12.1 Hz, H-4a), 2.37 (1H, m, H-4-Heq) and 1.62 (1H, ddd, *J =* 12.5, 12.0 and 11.8 Hz, H-4-Hax), 2.32 (1H, dd, *J =* 13.3 and 4.1 Hz, H-1-Heq) and 1.38 (1H, ddd, *J =* 12.3, 12.0 and 4.3 Hz, H-1-Hax), 2.05 (1H, dddd, *J =* 12.3, 12.2, 11.0, and 4.0 Hz, H-1a), 1.30 (3H, s, H-11). ¹³C NMR (125 MHz, CD₃OD): δ 202.0 (C-10), 166.7 (C-7), 165.0 (C-5), 150.5 (C-9a), 103.7 (C-8), 99.1 (C-6), 74.3 (C-3), 71.7 (C-9), 70.2 (C-2), 48.3 (C-4a), 41.8 (C-1a), 40.9 (C-1), 29.3 (C-4), 25.7 (C-11).

**Figure S2.** ^1^H-NMR spectrum (300 MHz; CDCl_3_) of compound **1**


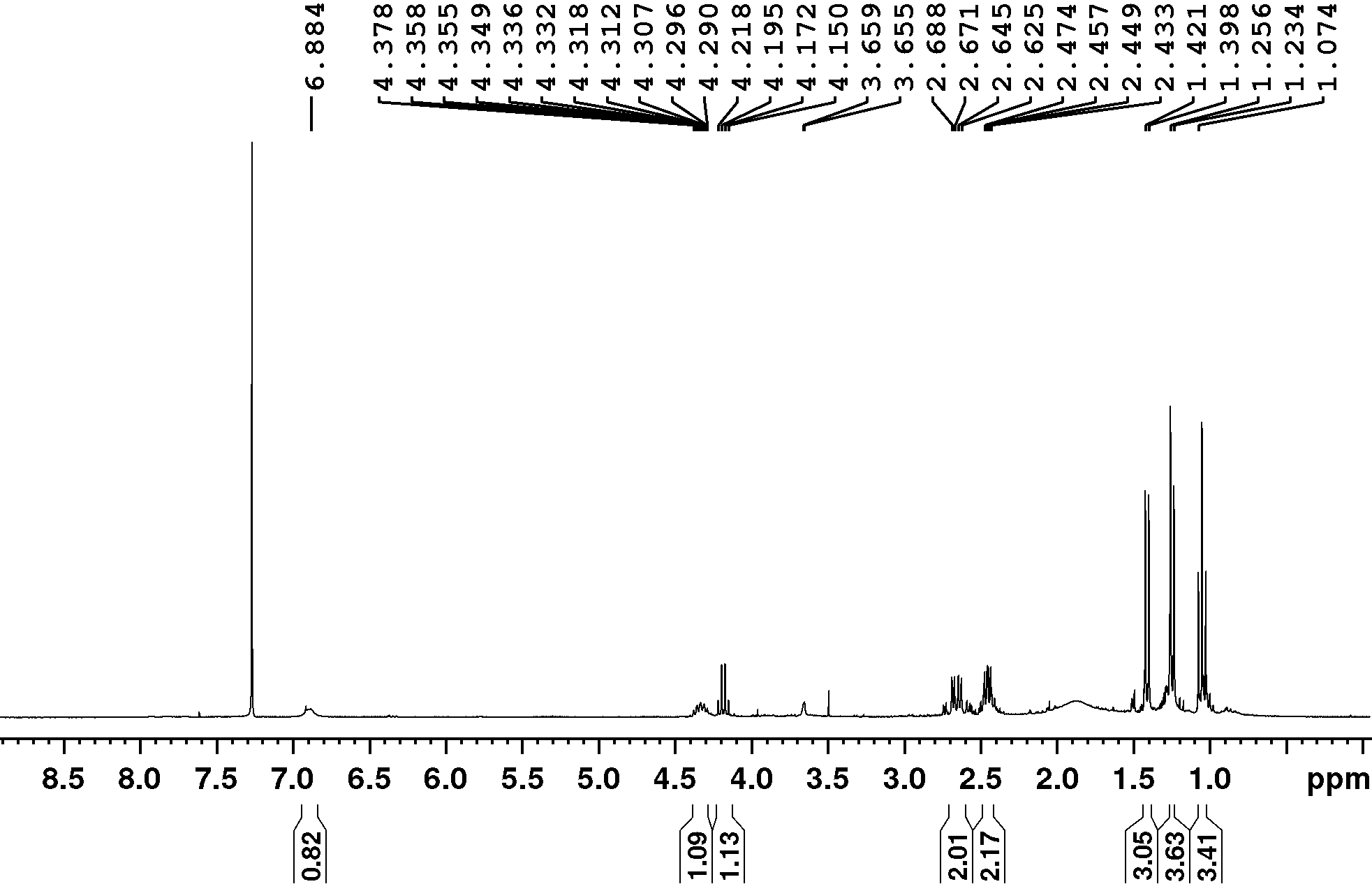


**Figure S3.** Expansions of the ¹H NMR spectrum (300 MHz, CDCl₃) of compound **1**


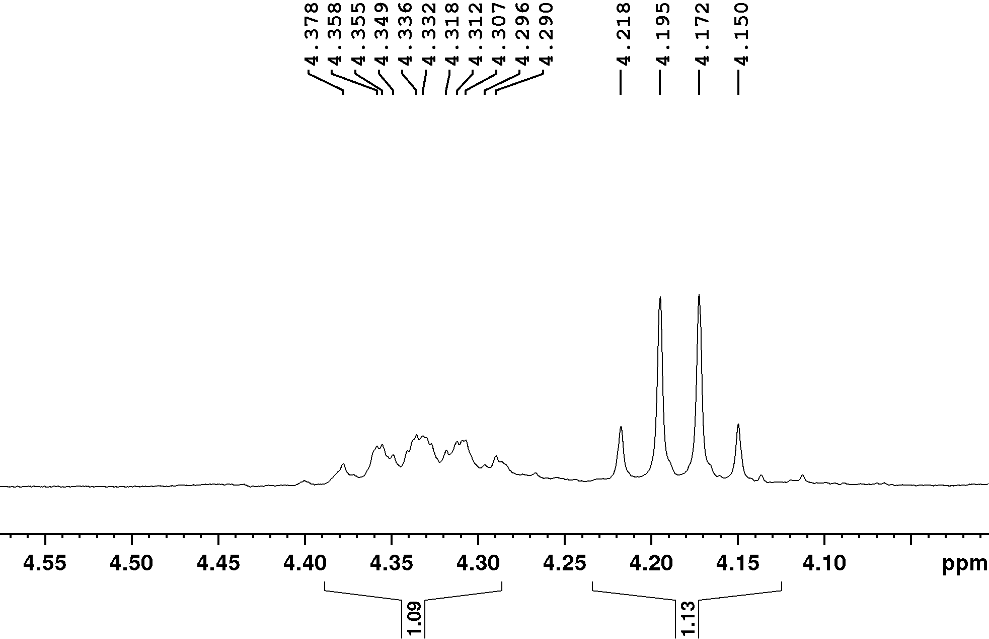


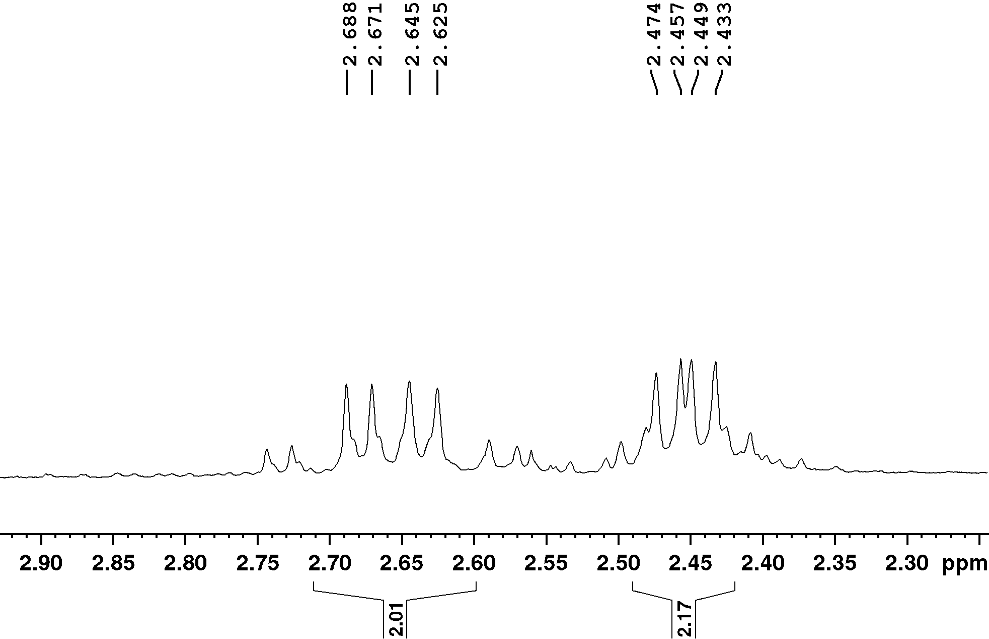


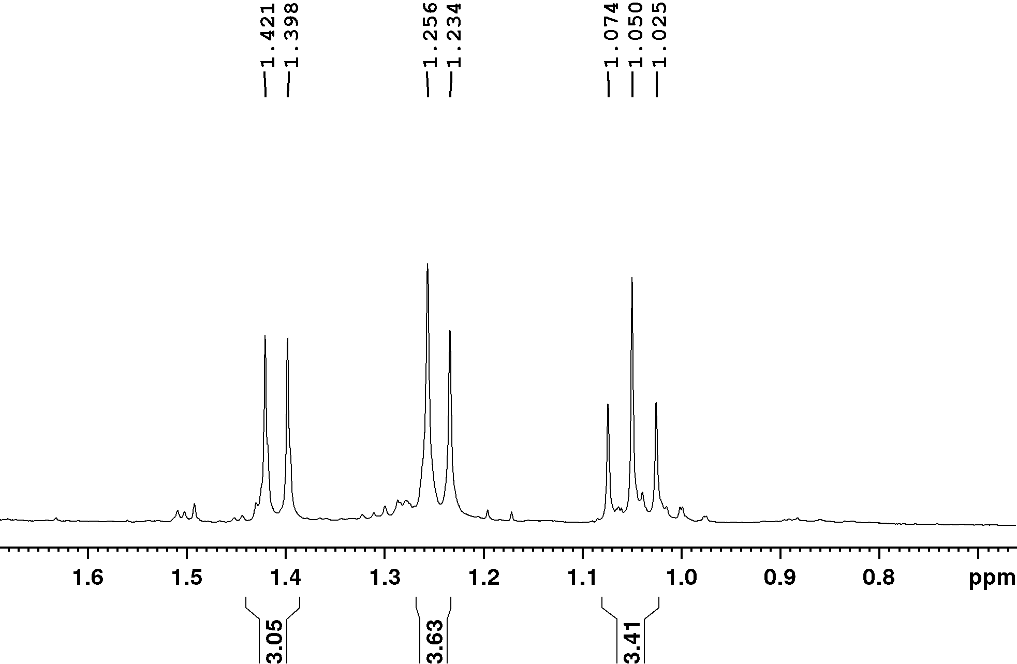


**Figure S4.** ^13^C-NMR spectrum (75,5 MHz; CDCl_3_) of compound **1**

**
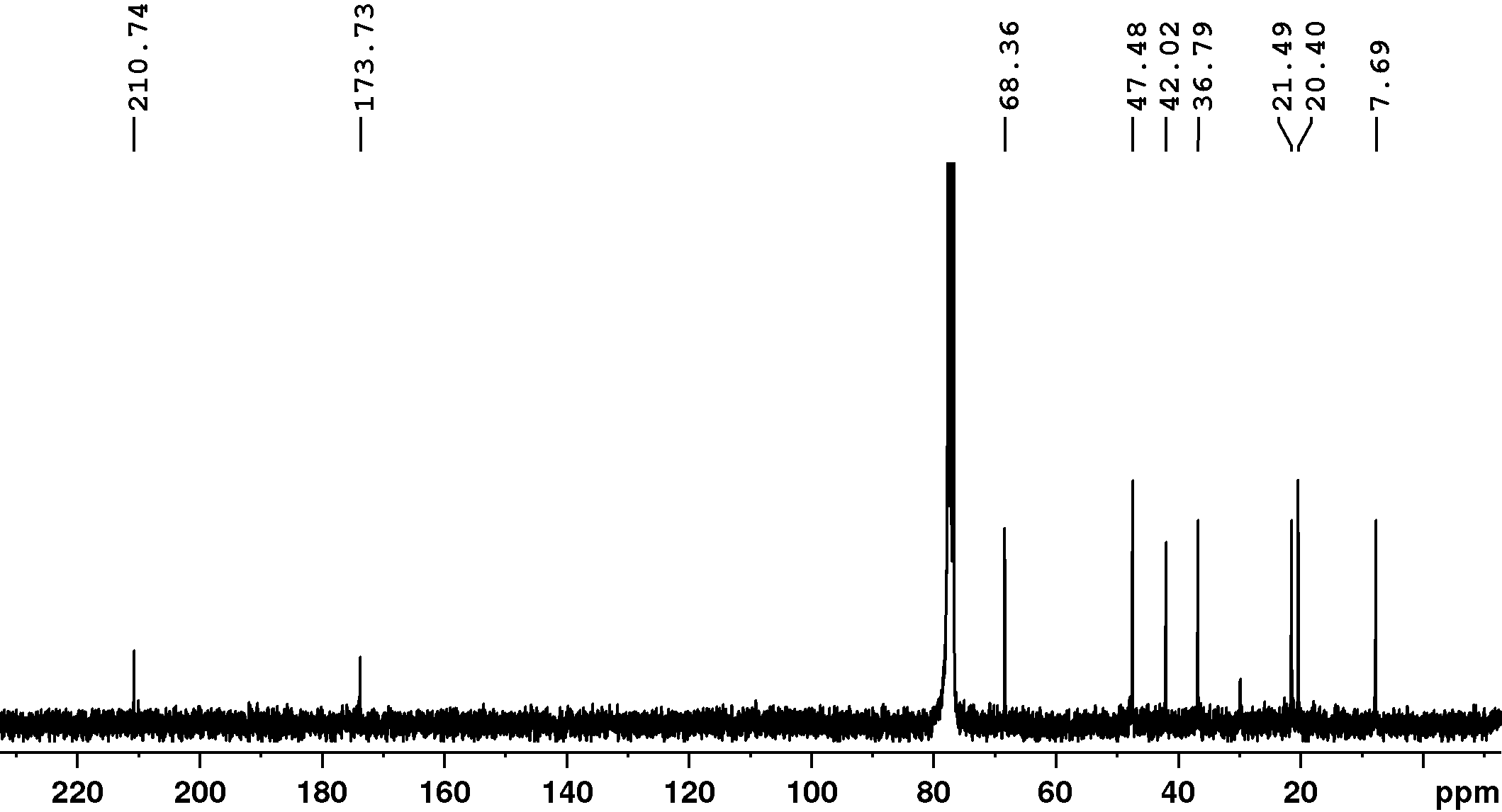
**

**Figure S5.** COSY spectrum (300 MHz, CDCl₃) of compound **1**

**
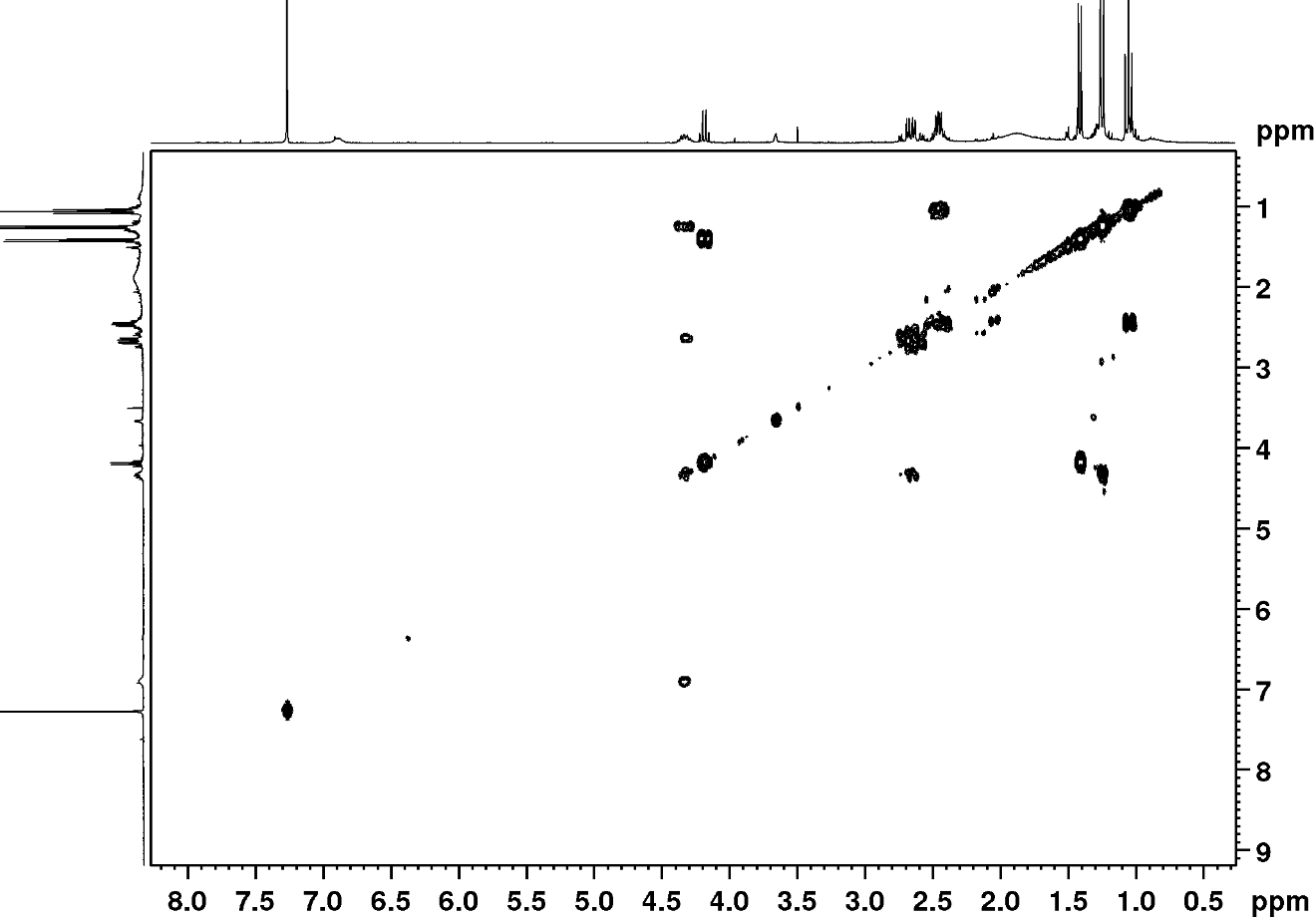
**

**Figure S6.** Expansion of the COSY spectrum (300 MHz, CDCl₃) of compound **1**

**
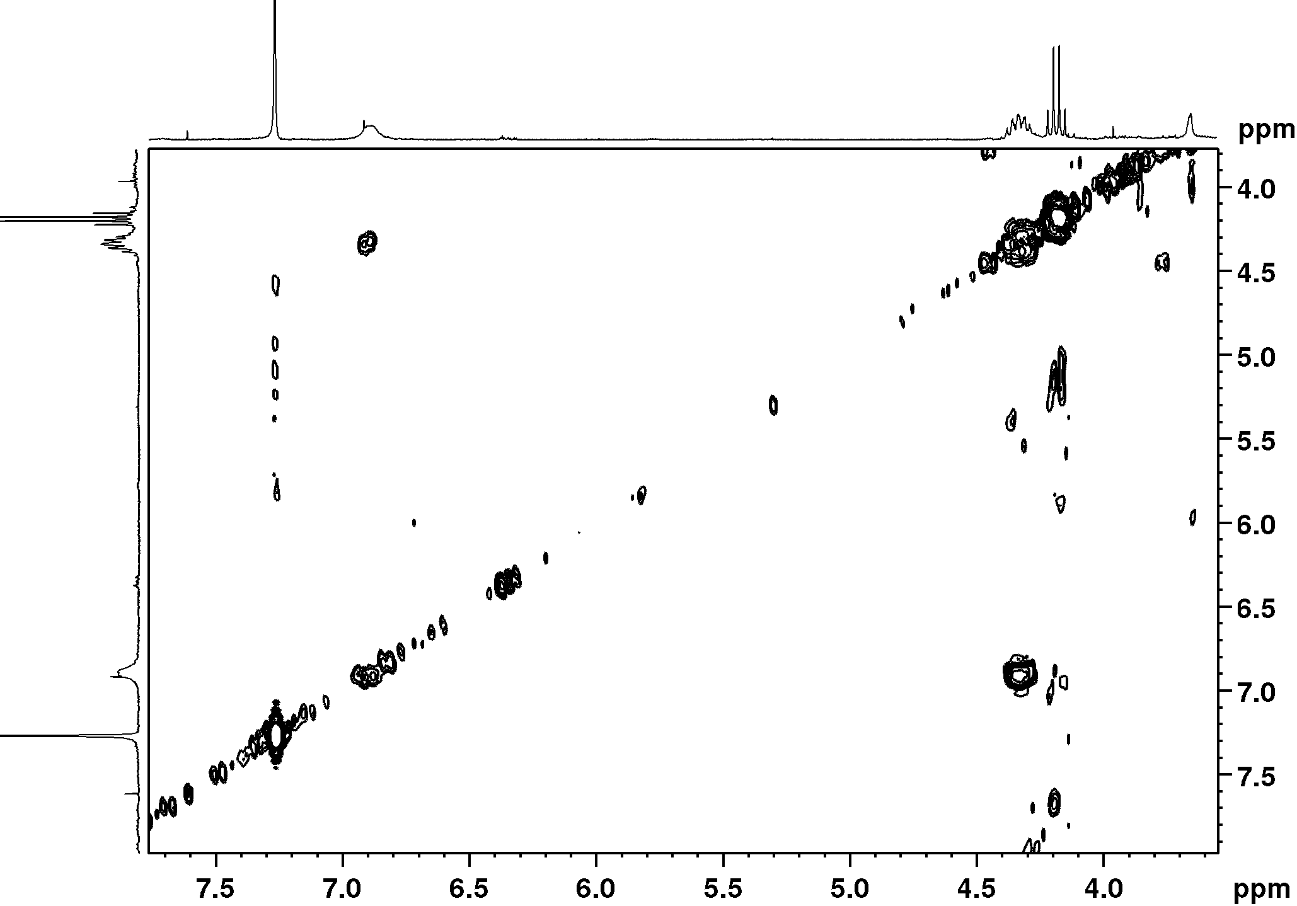
**

**Figure S7.** Expansion of the COSY spectrum (300 MHz, CDCl₃) of compound **1**

**
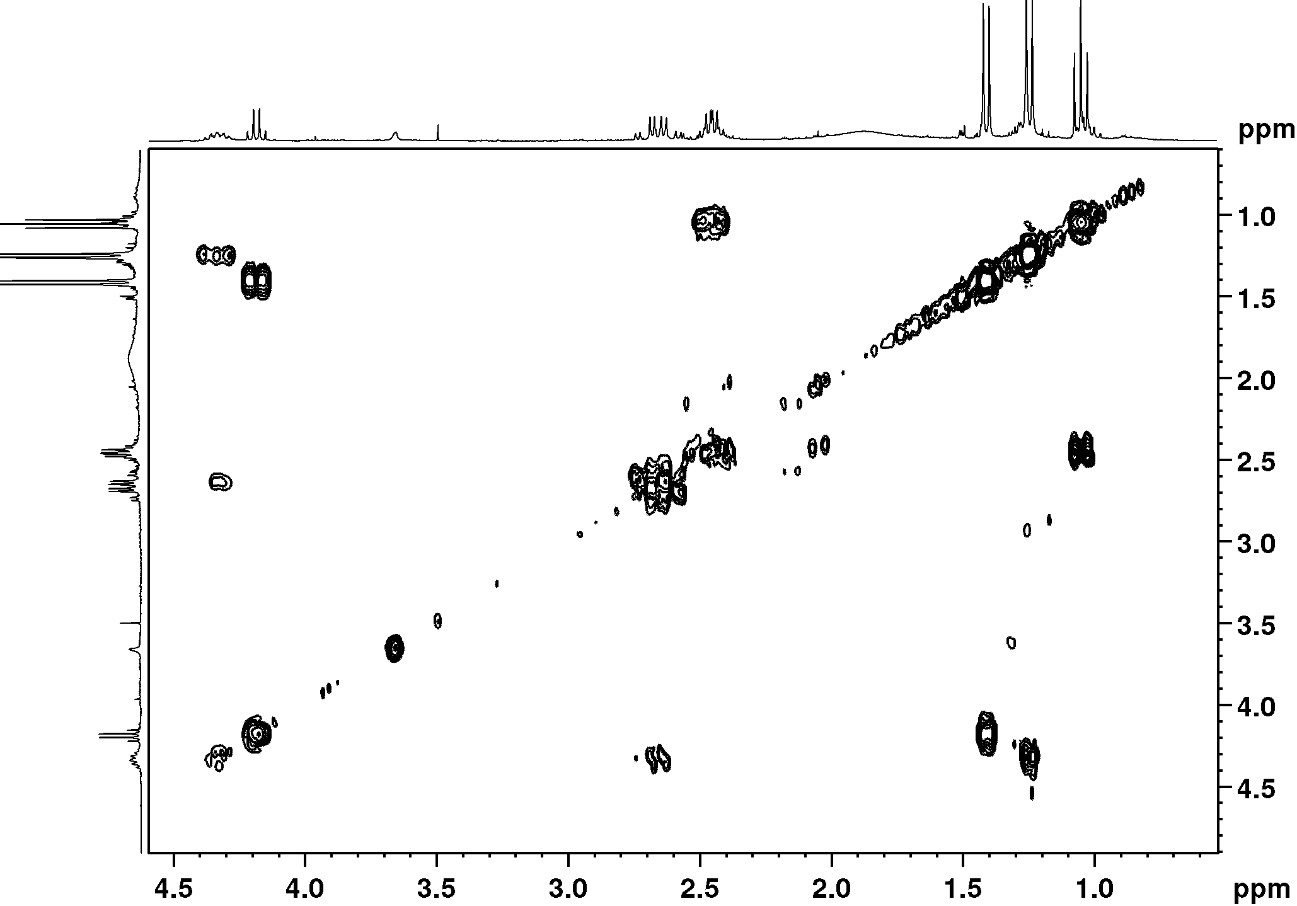
**

**Figure S8.** HSQC spectrum (300 e 75,5 MHz; CDCl_3_) of compound **1
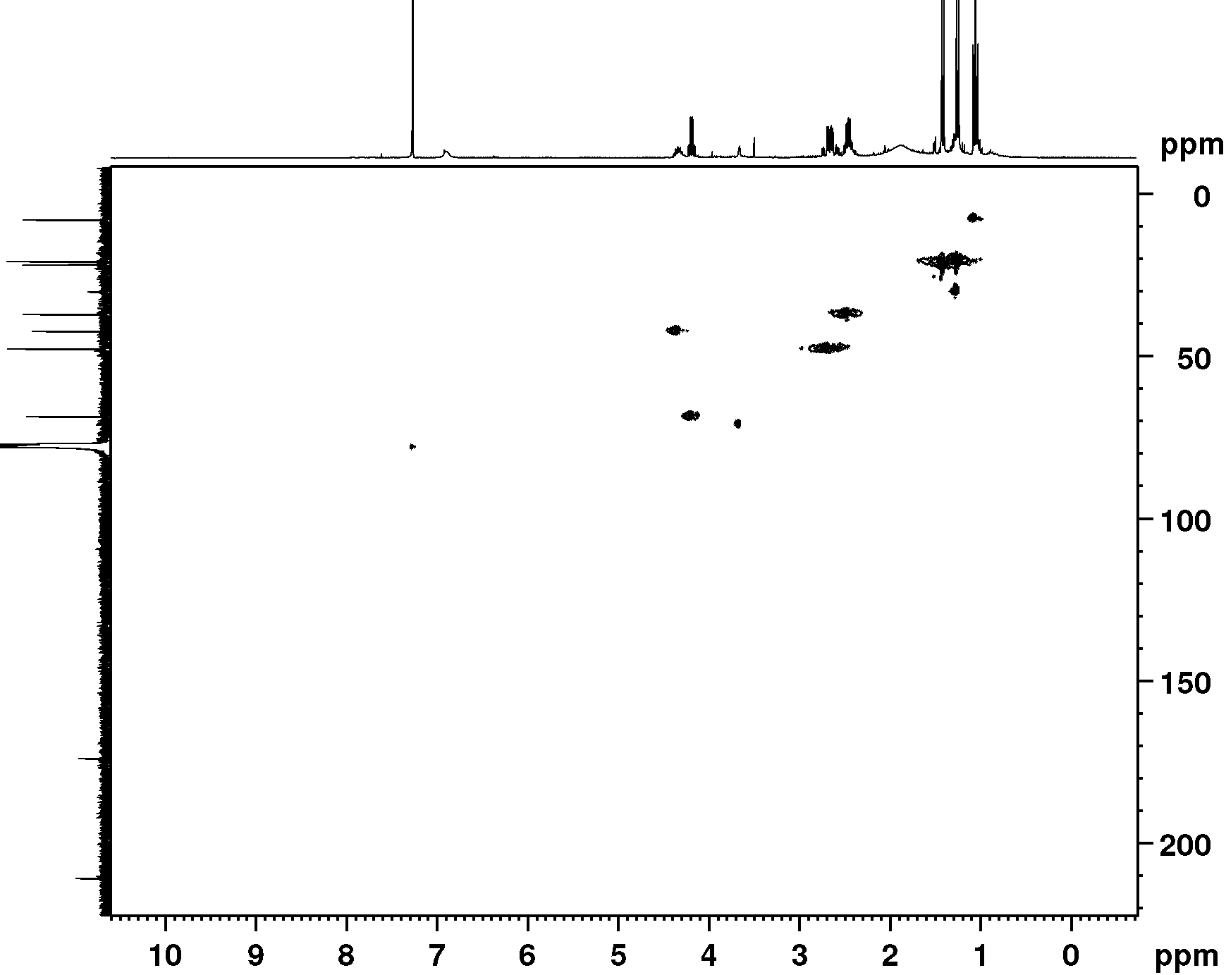
**

**Figure S9.** Expansion of the HSQC (300 e 75,5 MHz; CDCl_3_) for compound **1**

**
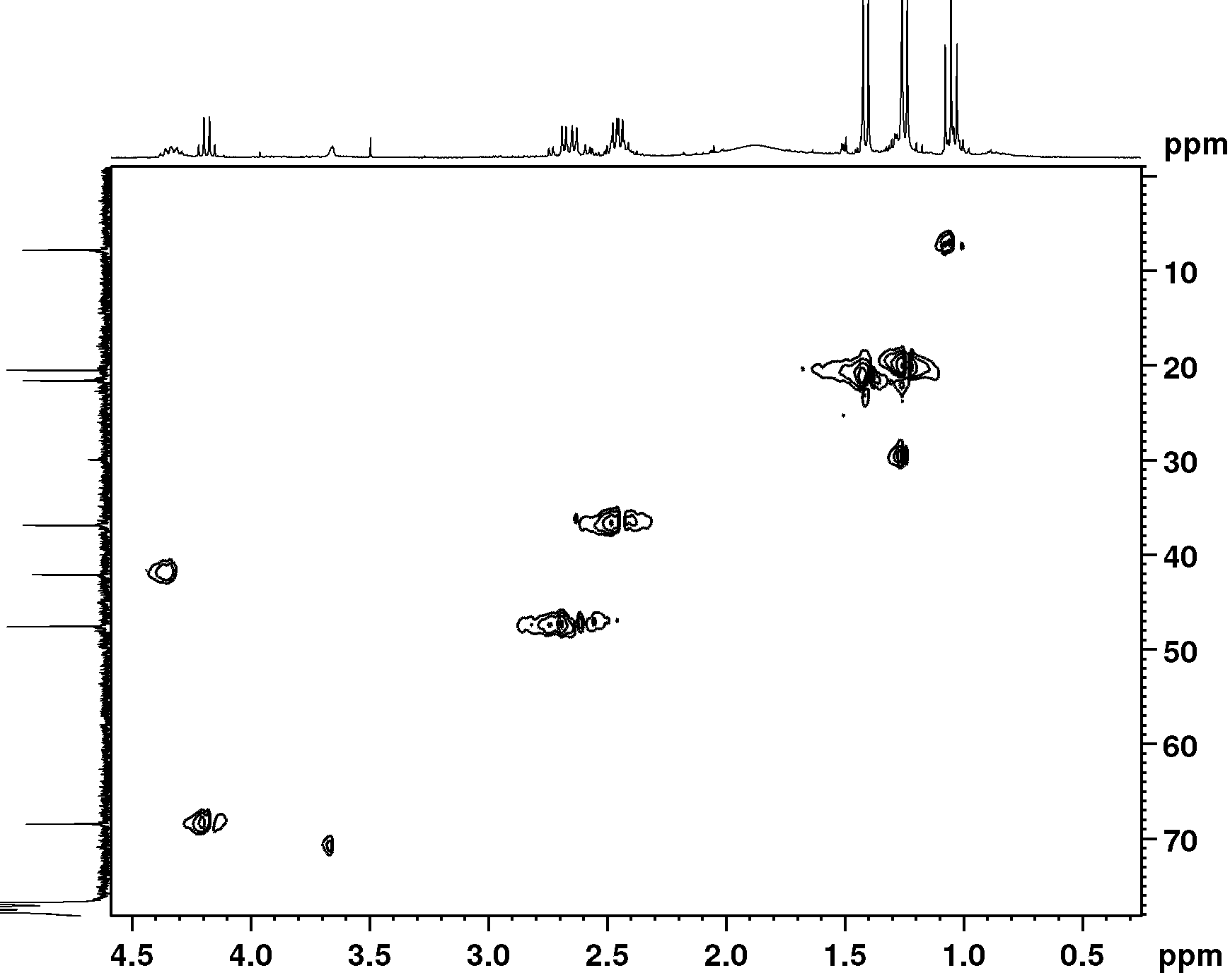
**

**Figure S10.** Expansion of the HMBC (300 e 75,5 MHz; CDCl_3_) for compound **1**


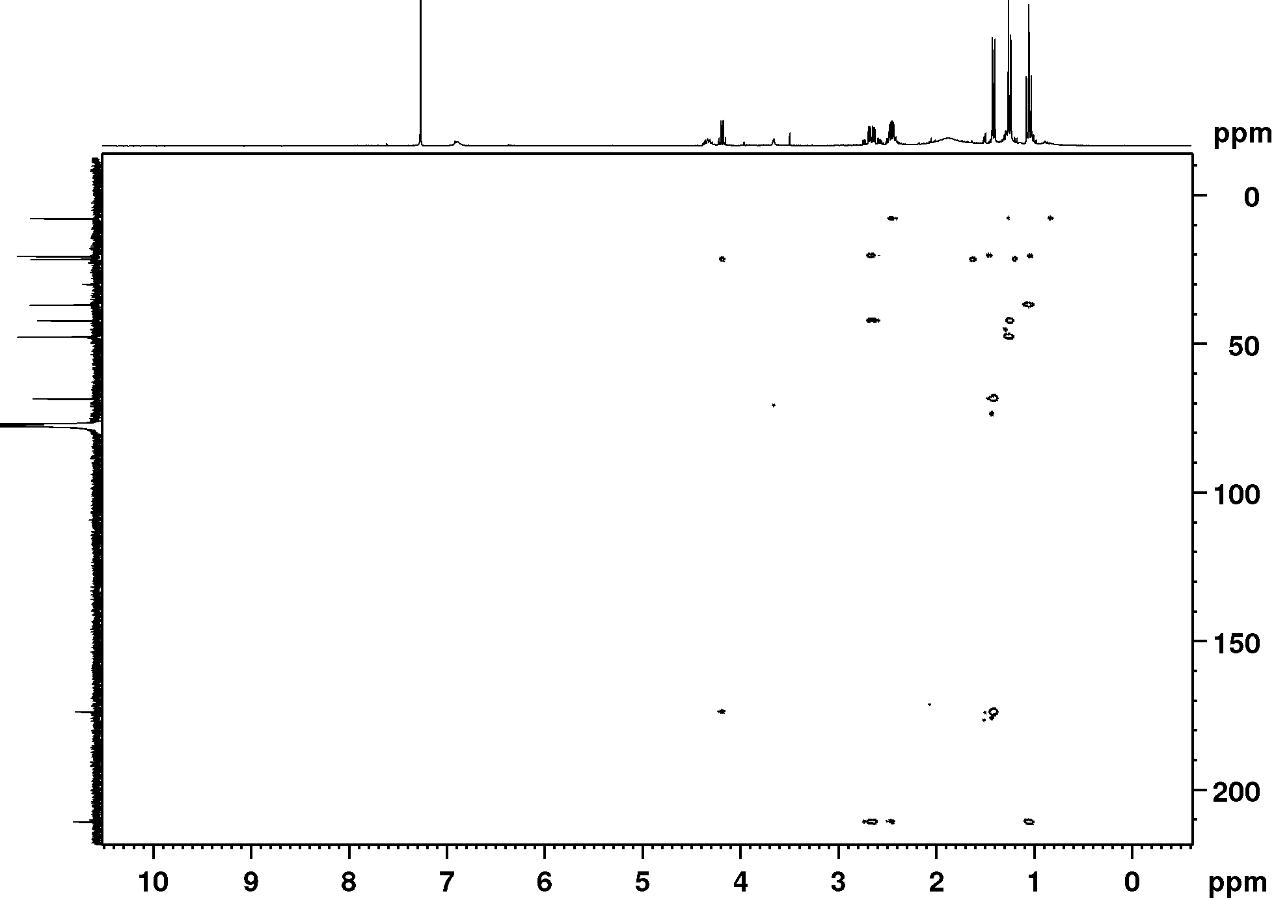


**Figure S11.** Expansion of the HMBC (300 e 75,5 MHz; CDCl_3_) for compound **1**


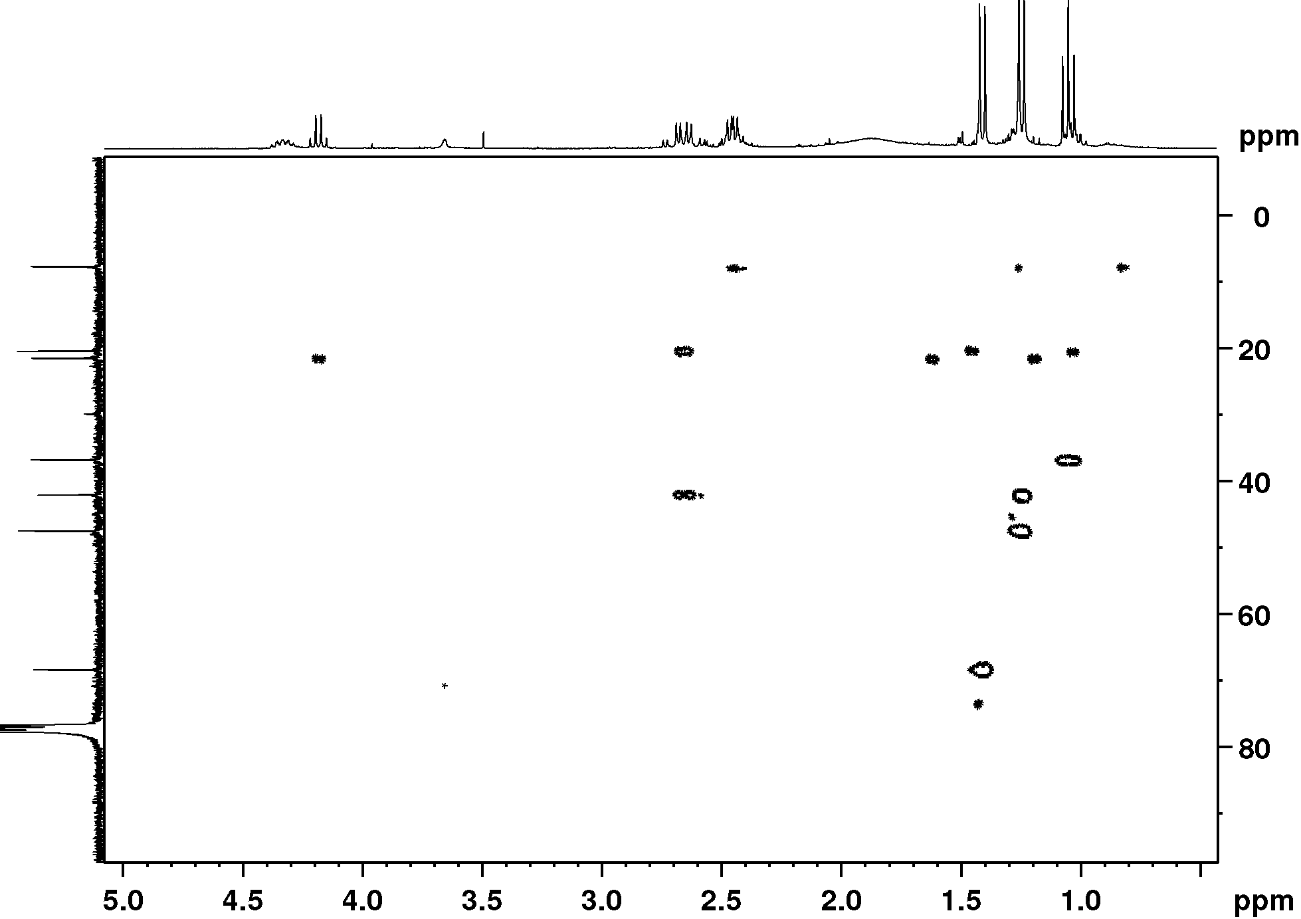


**Figure S12**. (+)HRESIMS/MS spectrum of compound **1**

**Figure S13**. ^1^H-NMR spectrum (300 MHz; CDCl_3_) of compounds **2** and **3**


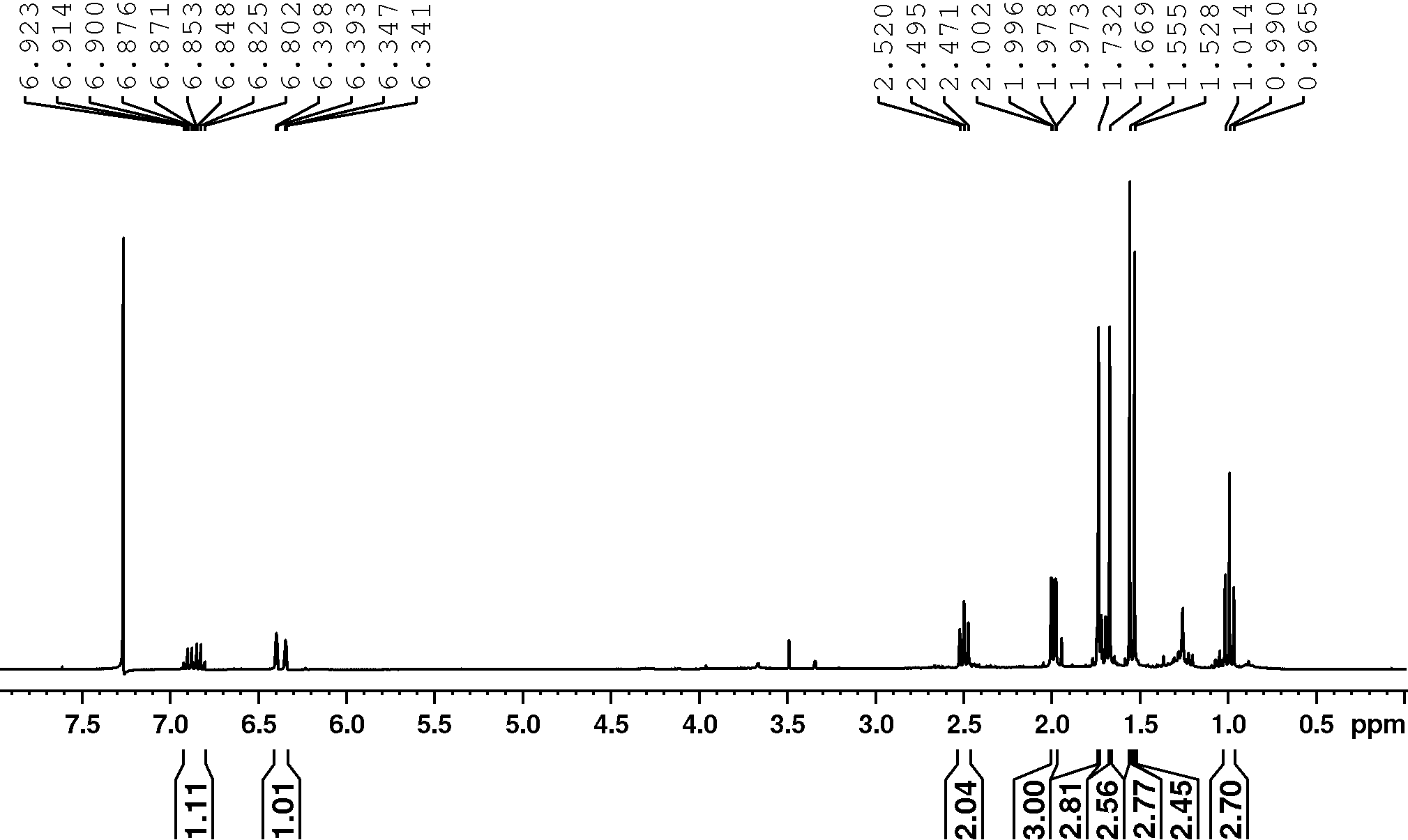


**Figure S14.** Expansion of the ^1^H NMR (300 MHz; CDCl3) for compounds **2** and **3**


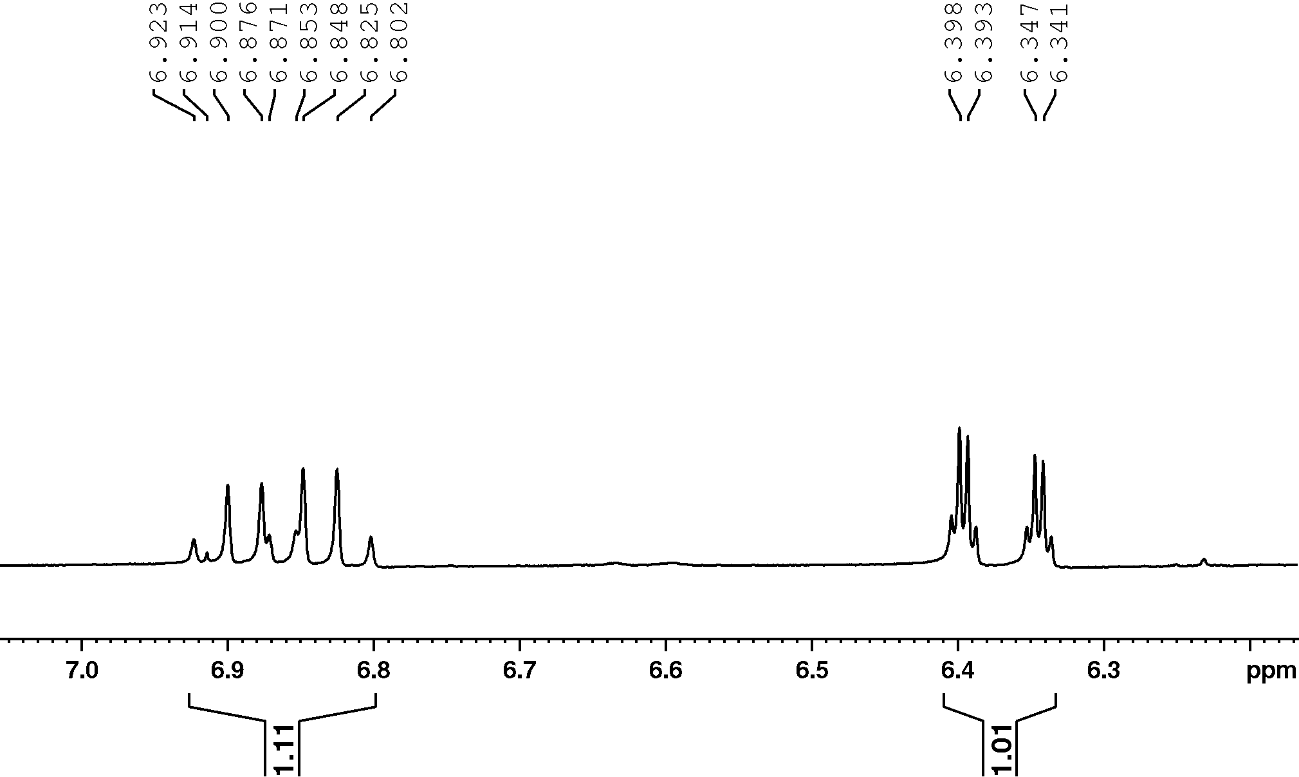


**Figure S15.** Expansion of the ¹H NMR spectrum (300 MHz, CDCl₃) of compounds 2 and 3.


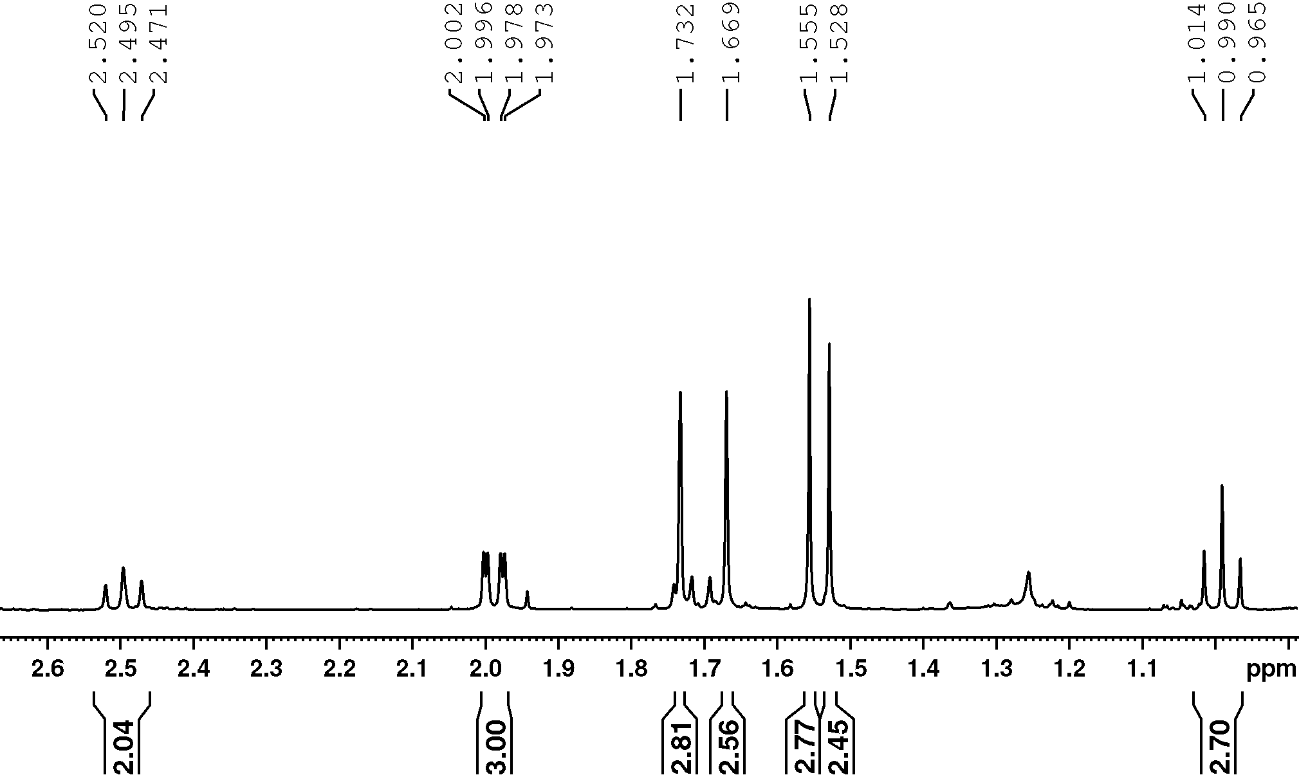
.

**Figure S16.** ^13^C-NMR spectrum (75,5 MHz; CDCl_3_) of compounds **2** and **3**


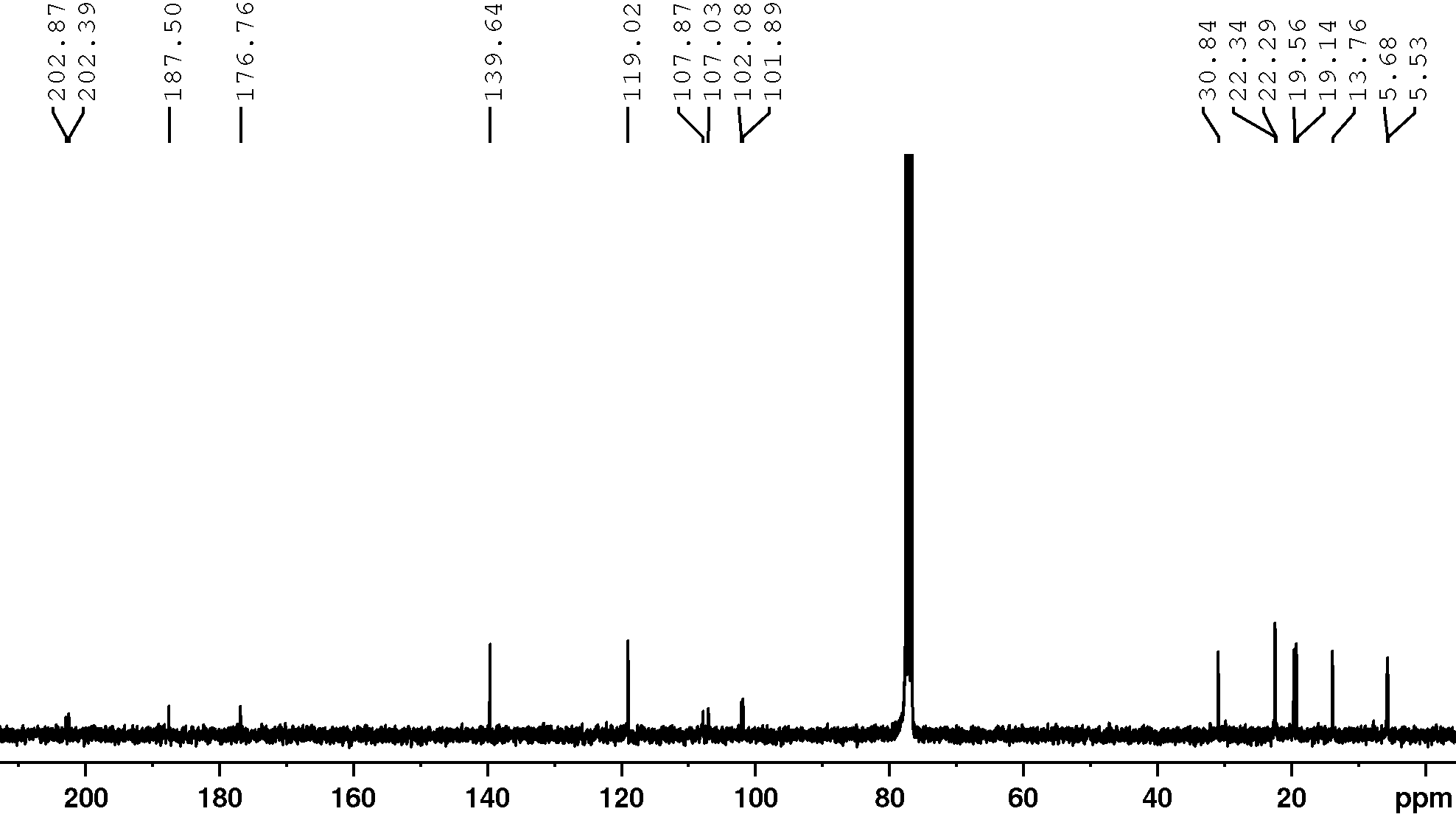


**Figure S17**. (+)HRESIMS/MS spectrum of compound **2**

**Figure S18**. (+)HRESIMS/MS spectrum of compound **3**

**Figure S19.** ^1^H-NMR spectrum (300 MHz; CD_3_OD) of compound **4**


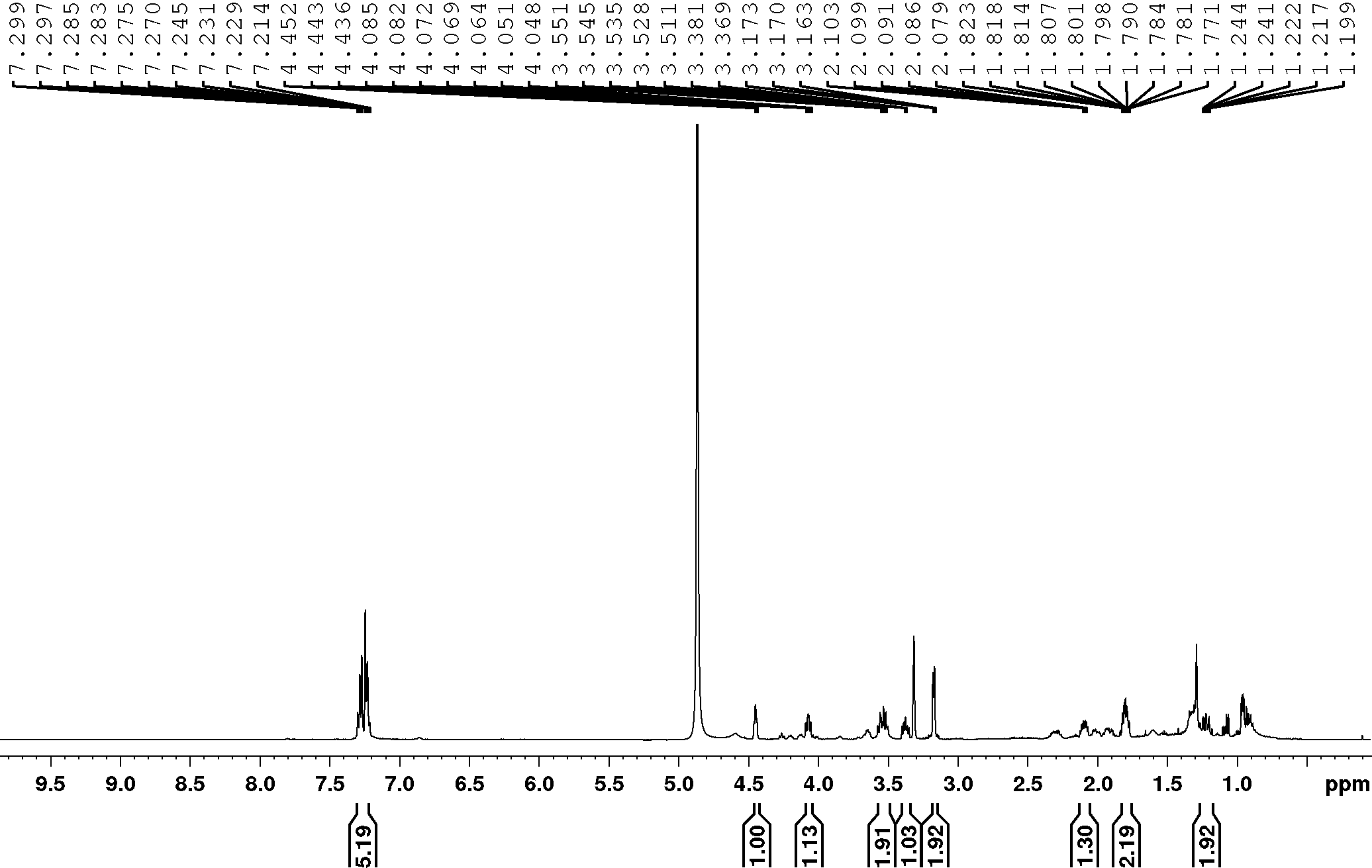


**Figure S20.** ^13^C-NMR spectrum (125 MHz; CD_3_OD) of compound **4**


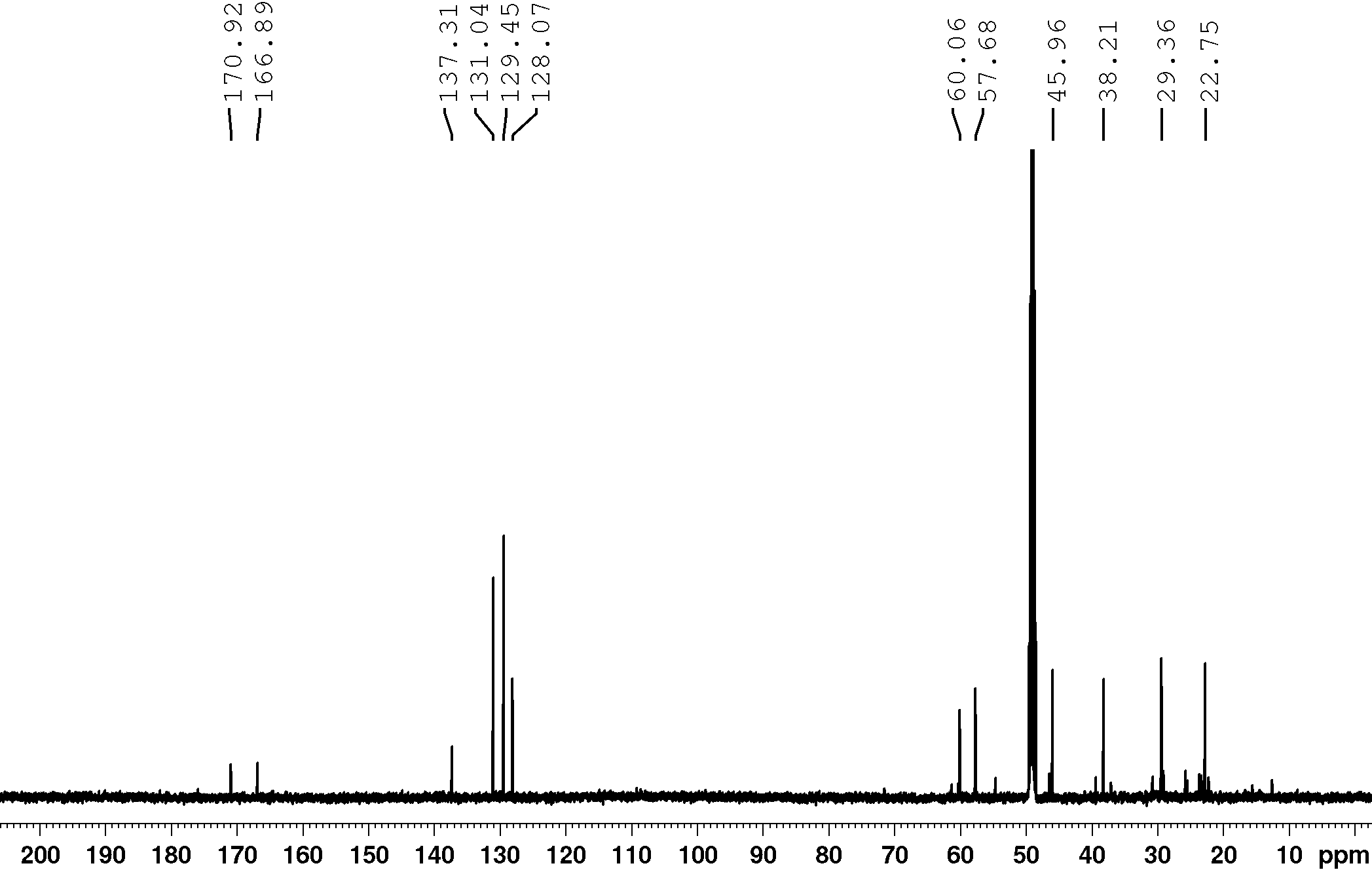


**Figure S21.** ^1^H-NMR spectrum (300 MHz; CD_3_OD) of compound **5**


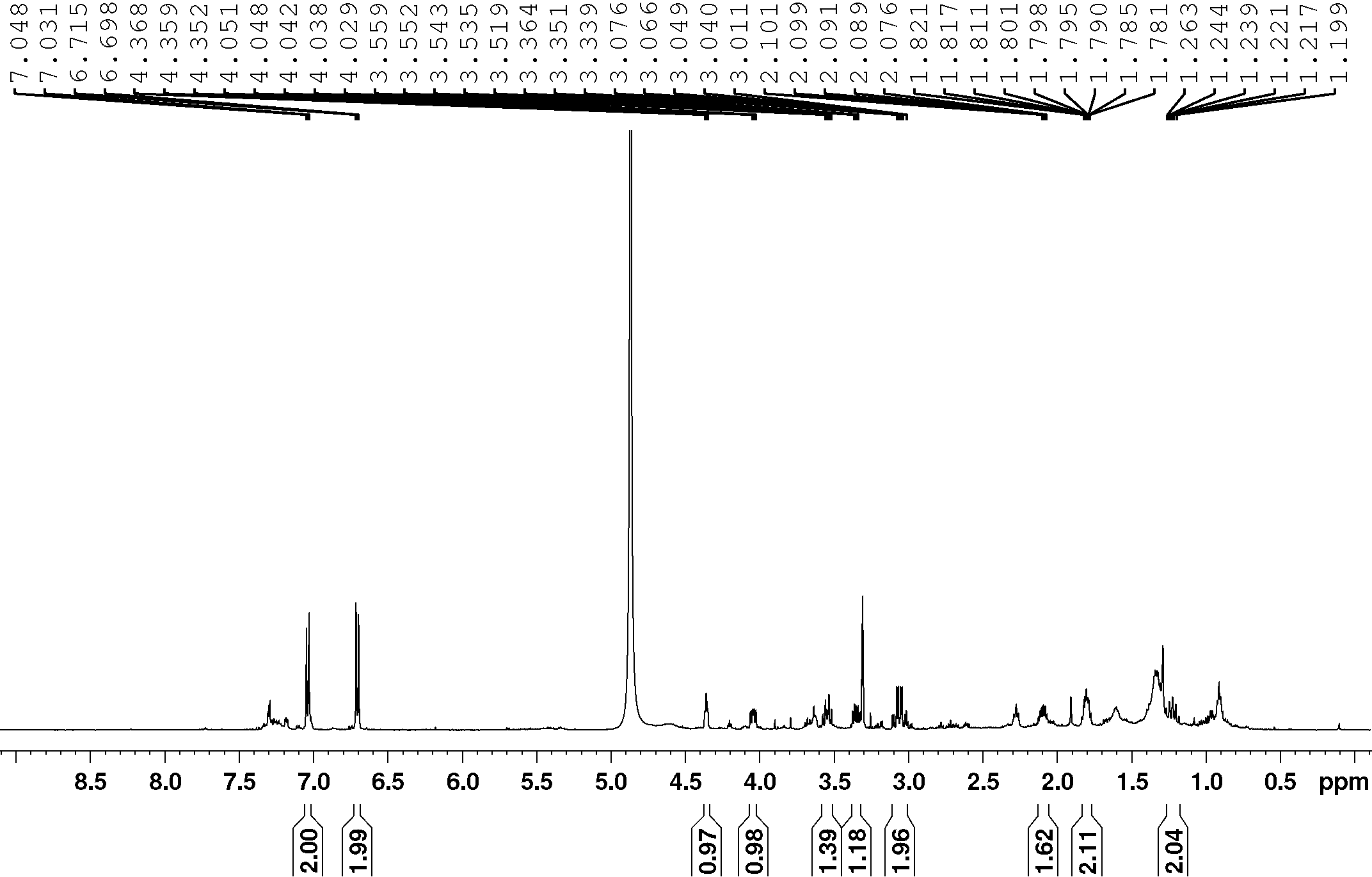


**Figure S22.** ^13^C-NMR spectrum (125 MHz; CD_3_OD) of compound **5**


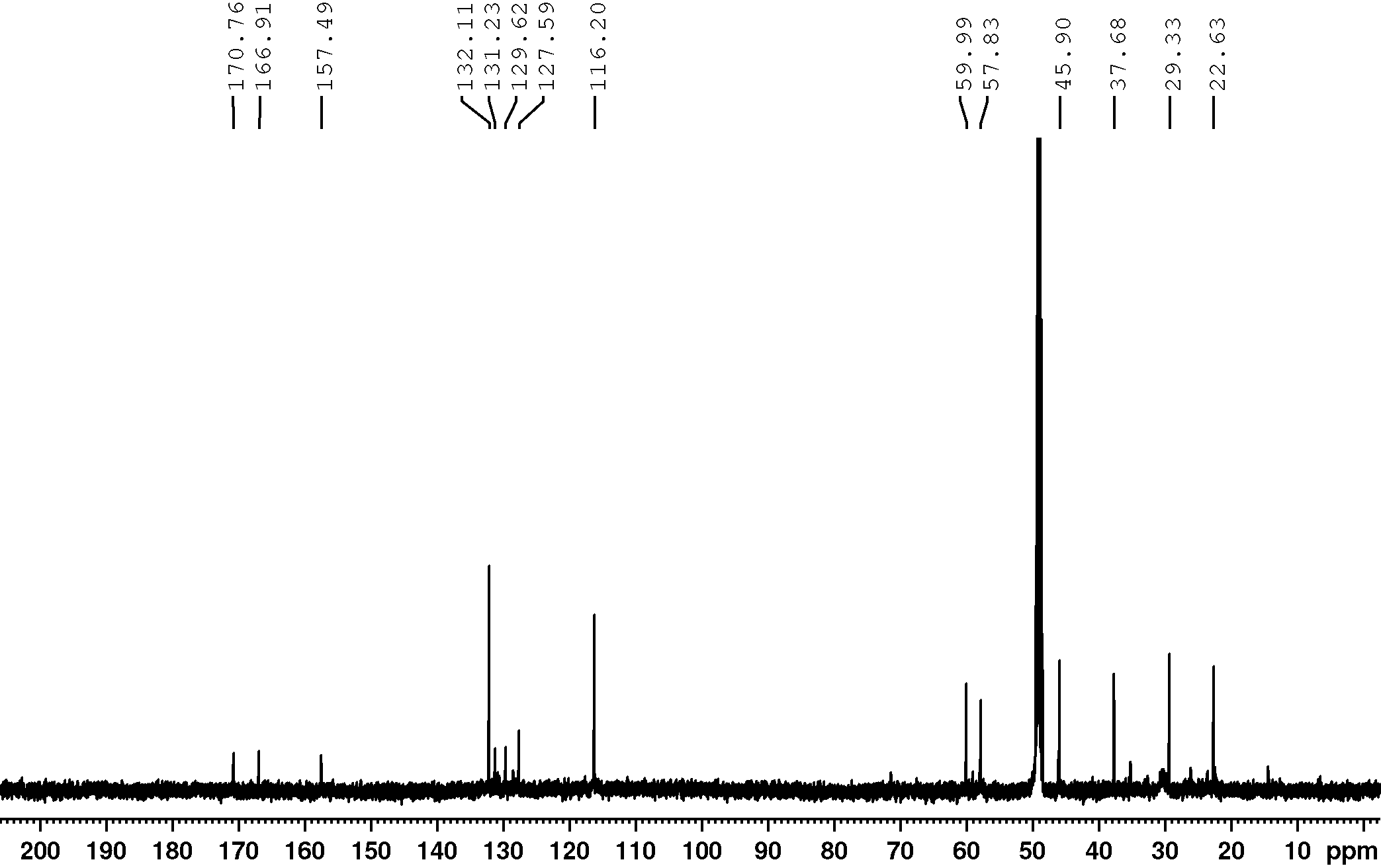


**Figure S23**. (+)HRESIMS/MS spectrum of compound **4**

**Figure S24**. (+)HRESIMS/MS spectrum of compound **5**

**Figure S25.** ^1^H-NMR spectrum (500 MHz; CD_3_OD) of compound **6**


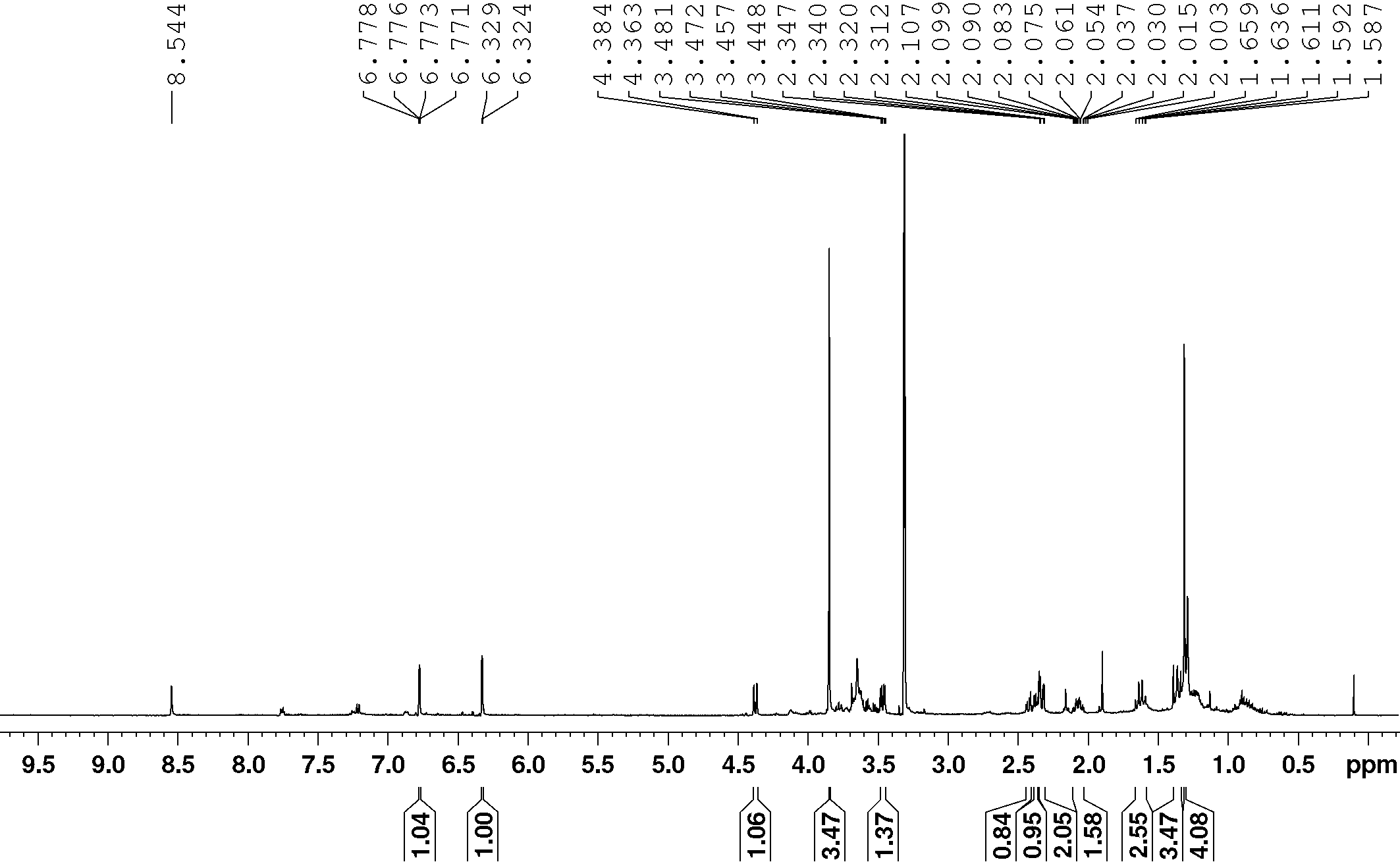


**Figure S26.** Expansion of the ¹H NMR spectrum (500 MHz, CD_3_OD) of compound **6**


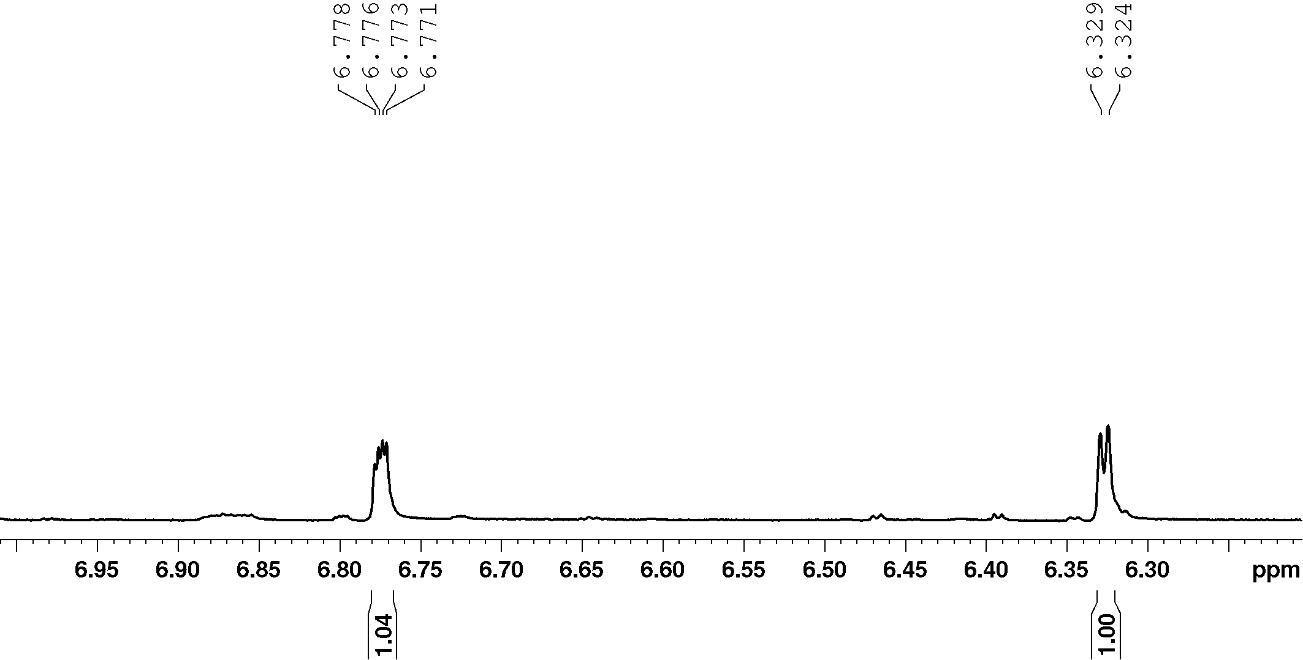


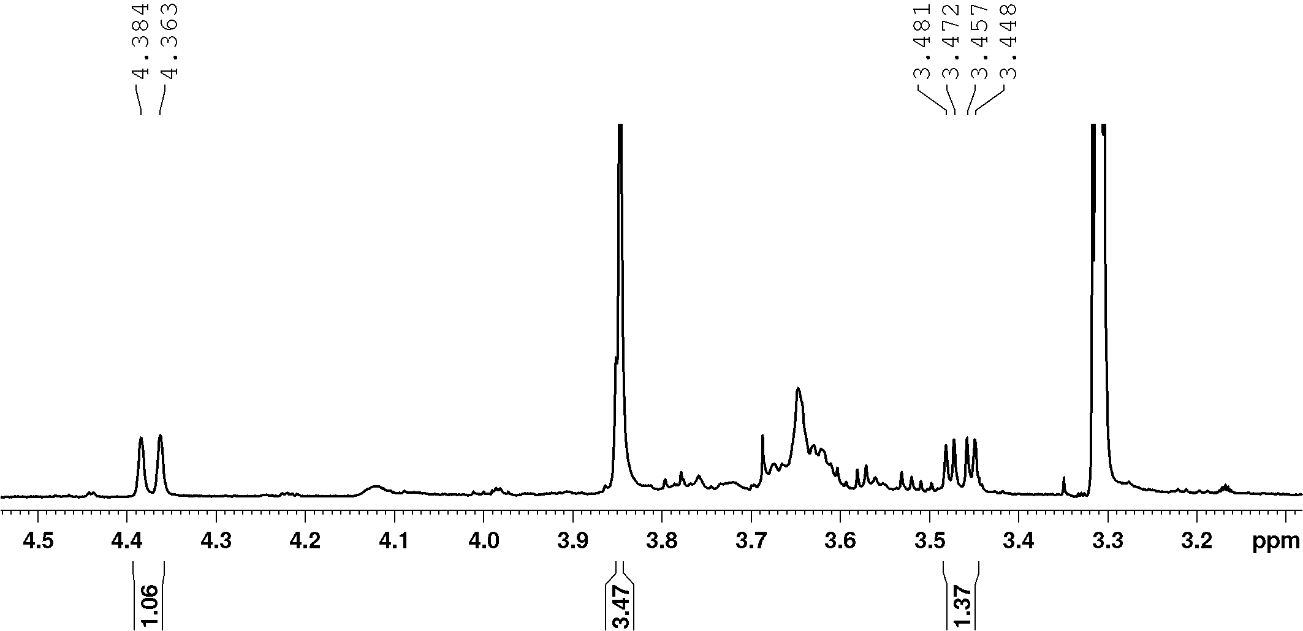


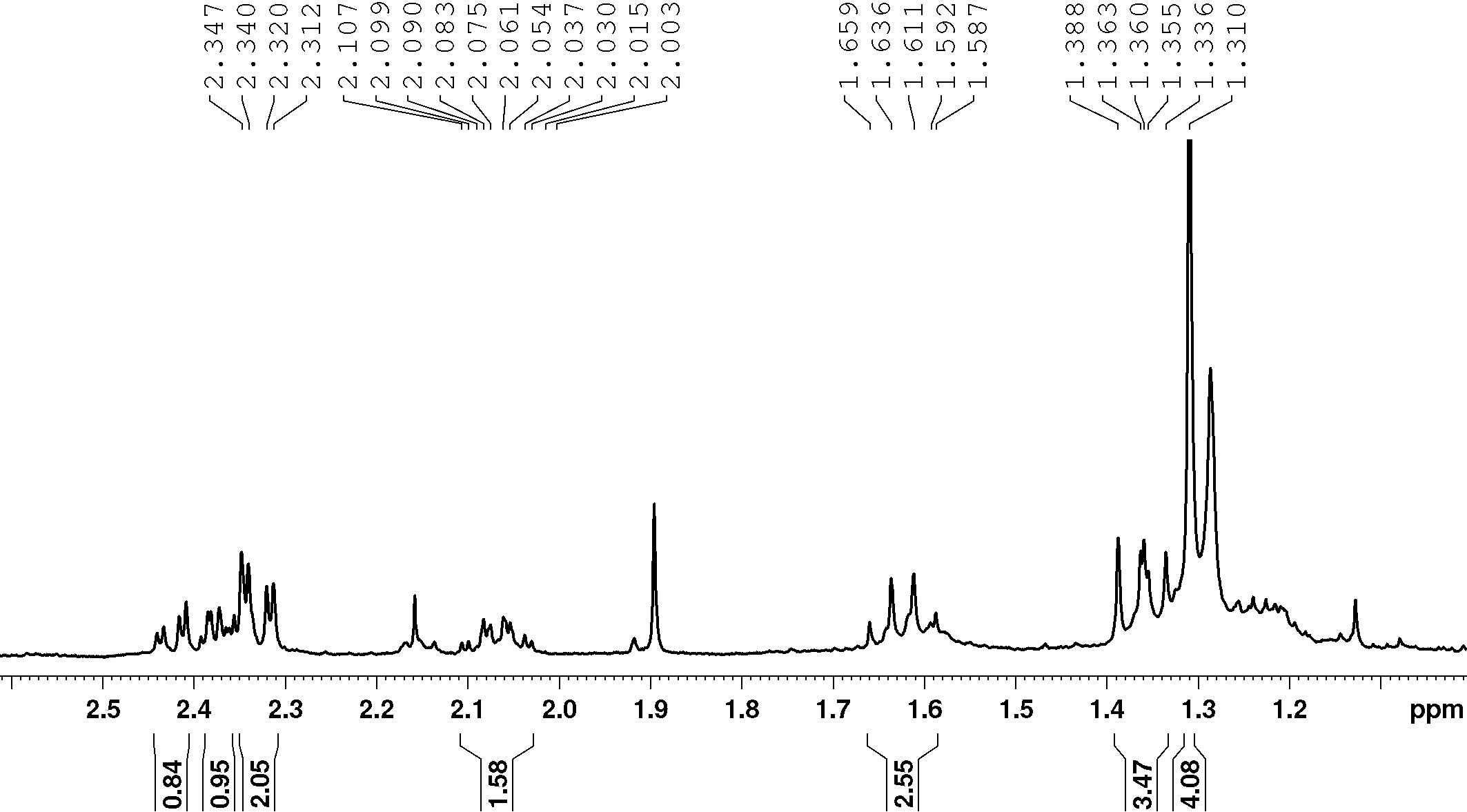


**Figure S27.** COSY spectrum (500 MHz; CD_3_OD) for compound **6**


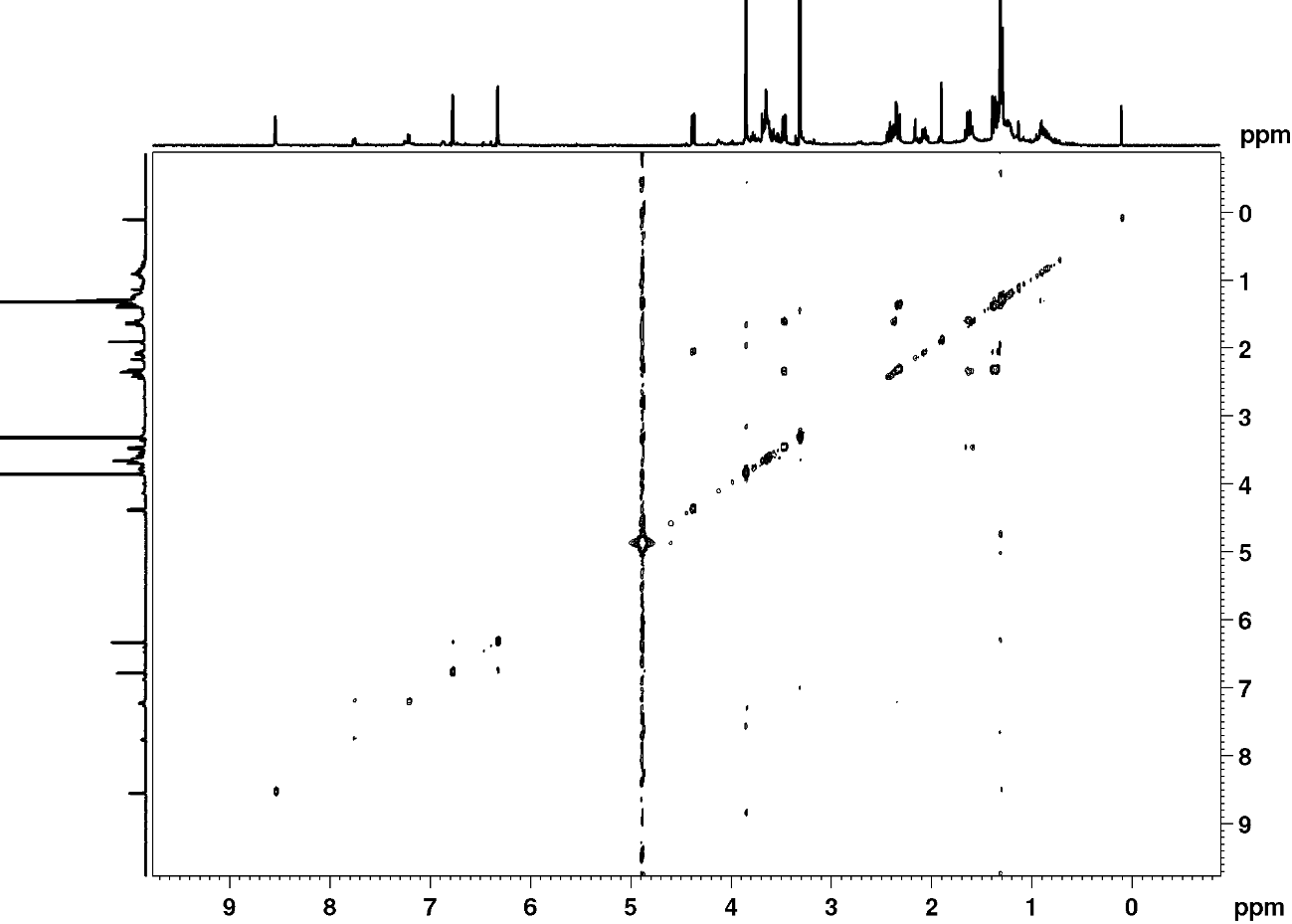


**Figure S28.** HSQC spectrum (500 e 125 MHz; CD_3_OD) of compound **6**


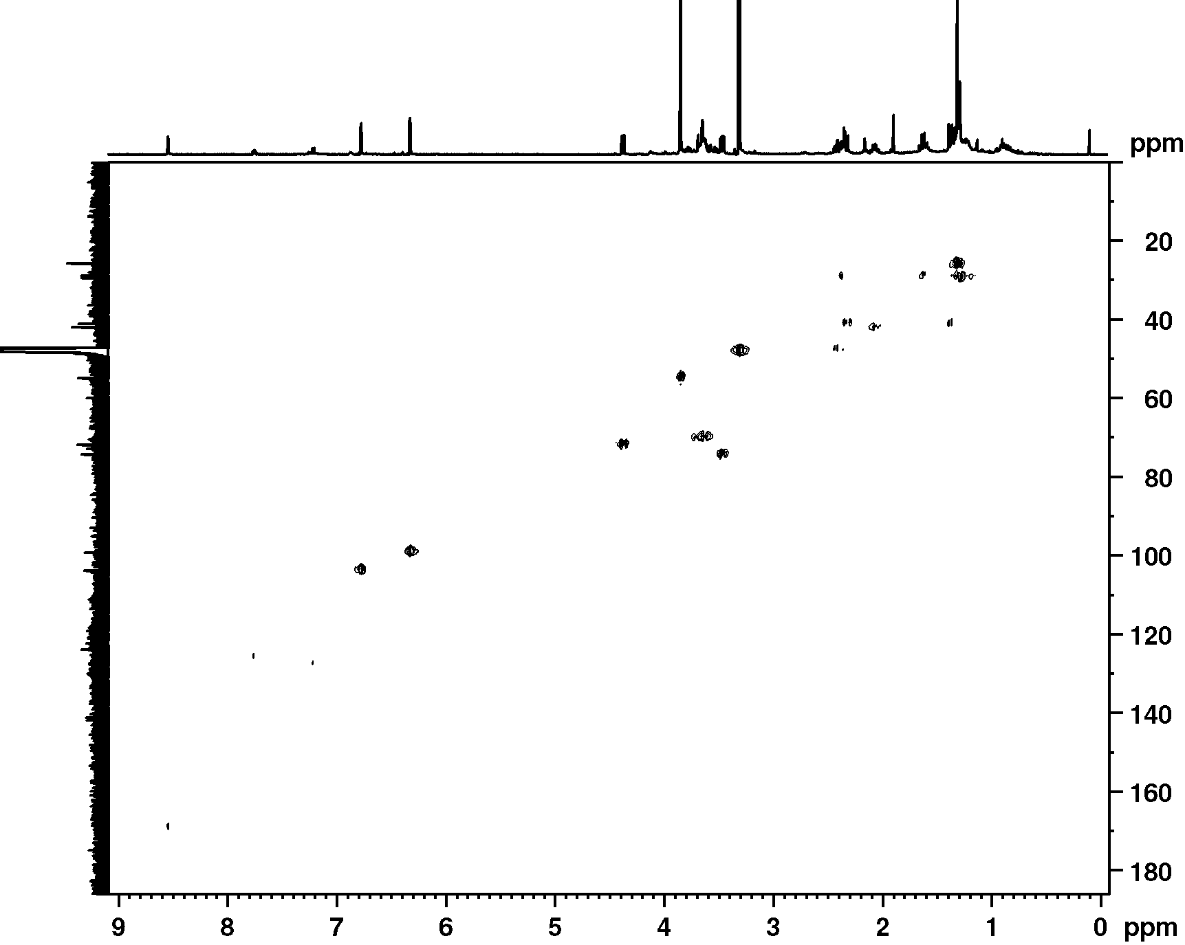


**Figure S29.** HMBC spectrum (500 e 125 MHz; CD_3_OD) of compound **6**


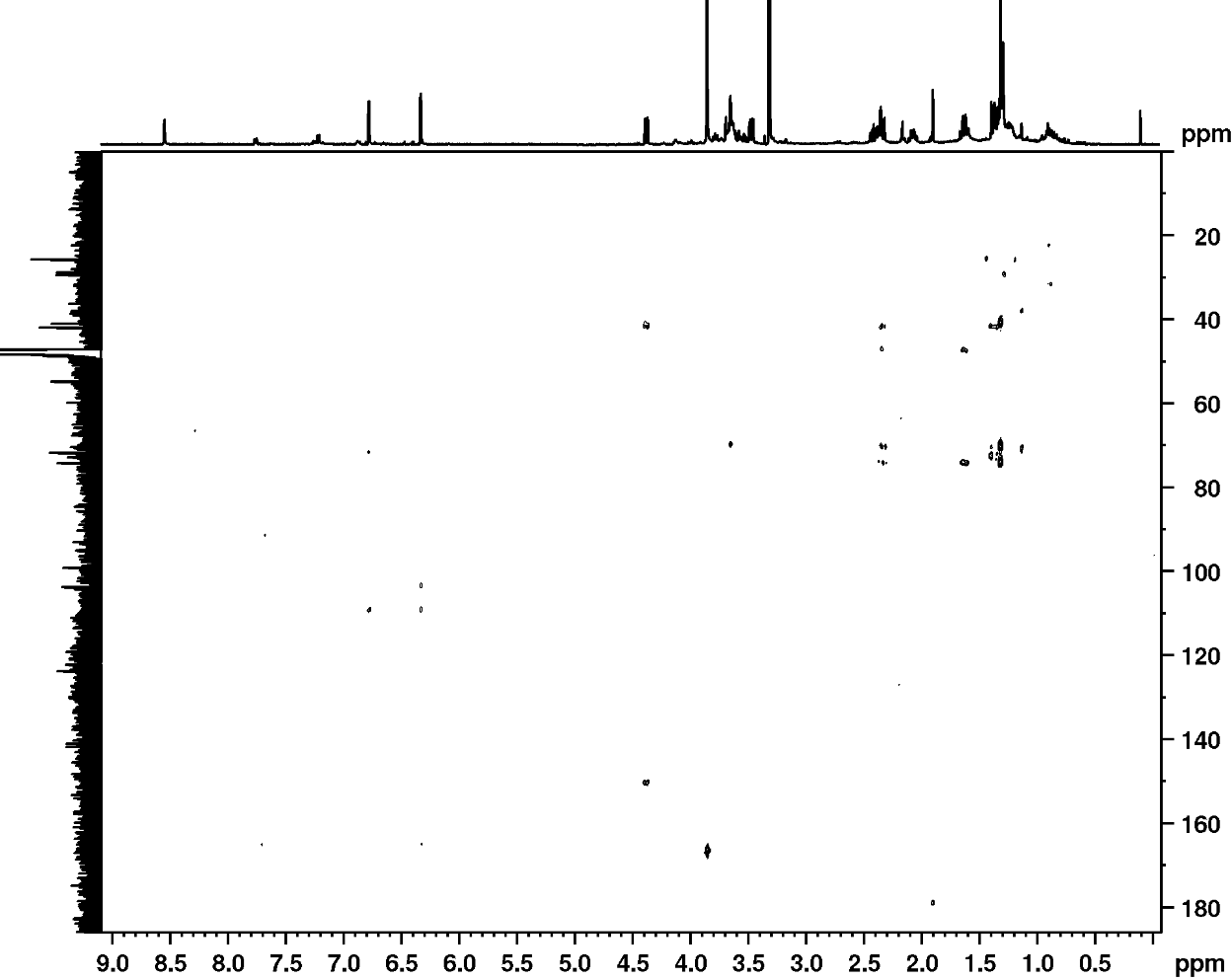


**Figure S30**. (-)HRESIMS/MS spectrum of compound **6**

**Table S1** - Pharmacokinetic parameters calculated using SwissADME data base for compound **1**

| **Parameters** | **Molecule**  **Compound 1** |
| --- | --- |
| Formula | C_9_H_17_NO_3_ |
| MW | 187,24 |
| #Heavy atoms | 13 |
| #Aromatic heavy atoms | 0 |
| Fraction Csp3 | 0,78 |
| #Rotatable bonds | 6 |
| #H-bond acceptors | 3 |
| #H-bond donors | 2 |
| MR | 49,74 |
| TPSA | 66,4 |
| iLOGP | 1,48 |
| XLOGP3 | -0,15 |
| WLOGP | 0,24 |
| MLOGP | 0,03 |
| Silicos-IT Log P | 0,78 |
| Consensus Log P | 0,48 |
| ESOL Log S | -0,51 |
| ESOL Solubility (mg/ml) | 57,8 |
| ESOL Solubility (mol/l) | 0,309 |
| ESOL Class | Very soluble |
| Ali Log S | -0,79 |
| Ali Solubility (mg/ml) | 30,4 |
| Ali Solubility (mol/l) | 0,162 |
| Ali Class | Very soluble |
| Silicos-IT LogSw | -1,32 |
| Silicos-IT Solubility (mg/ml) | 8,88 |
| Silicos-IT Solubility (mol/l) | 0,0474 |
| Silicos-IT class | Soluble |
| GI absorption | High |
| BBB permeant | No |
| Pgp substrate | No |
| CYP1A2 inhibitor | No |
| CYP2C19 inhibitor | No |
| CYP2C9 inhibitor | No |
| CYP2D6 inhibitor | No |
| CYP3A4 inhibitor | No |
| log Kp (cm/s) | -7,55 |
| Lipinski #violations | 0 |
| Ghose #violations | 0 |
| Veber #violations | 0 |
| Egan #violations | 0 |
| Muegge #violations | 1 |
| Bioavailability Score | 0,55 |
| PAINS #alerts | 0 |
| Brenk #alerts | 0 |
| Leadlikeness #violations | 1 |
| Synthetic Accessibility | 2,22 |

Pharmacokinetic parameters (http://www.swissadme.ch/index.php#) and target prediction (http://www.swisstargetprediction.ch/result.php?job=116917856&organism=Homo_sapiens) were calculated using SwissADMEdatabase as reported by Daina et al. (2017).

**Figure S31**- The Bioavailability Radar obtained using SwissADME data base for compound **1**


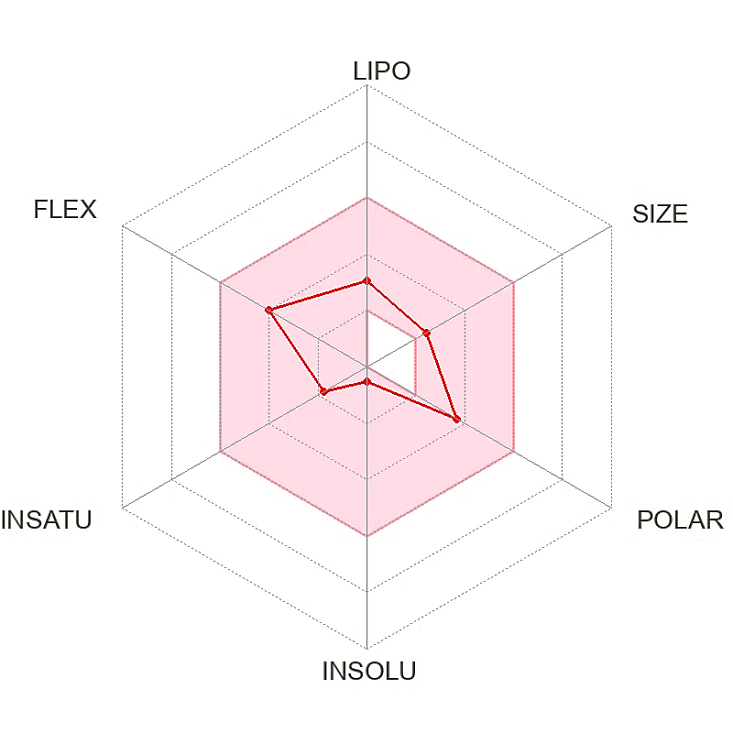


The pink area represents the optimal range for each properties.

Pharmacokinetic parameters (http://www.swissadme.ch/index.php#) and target prediction (http://www.swisstargetprediction.ch/result.php?job=116917856&organism=Homo_sapiens) were calculated using SwissADMEdatabase as reported by Daina et al. (2017).
